# All-optical multimodal mapping of single-cell-type-specific metabolic activities via REDCAT

**DOI:** 10.1038/s41592-026-03180-0

**Published:** 2026-08-27

**Authors:** Yajuan Li, Zhaojun Zhang, Archibald Enninful, Negin Farzad, Presha Rajbhandari, Hua Tian, Jungmin Nam, Xiaoyu Qin, Jorge Villazon, Anthony A. Fung, Hongje Jang, Zhiliang Bai, Nancy R. Zhang, Brent R. Stockwell, Rong Fan, Mina L. Xu, Zongming Ma, Lingyan Shi

**Affiliations:** 1 Shu Chien-Gene Lay Department of Bioengineering, University of California, University of California, San Diego, La Jolla, CA USA; 2 Department of Statistics and Data Science, Wharton School, University of Pennsylvania, Philadelphia, PA USA; 3 Department of Biomedical Engineering, Yale University, New Haven, CT USA; 4 Department of Chemistry, Columbia University, New York, NY USA; 5 Department of Biological Sciences, Columbia University, New York, NY USA; 6 Environmental and Occupational Health, Pitt Public Health, Pittsburgh, PA USA; 7 Children’s Neuroscience Institute, School of Medicine, Pittsburgh, PA USA; 8 Herbert Irving Comprehensive Cancer Center, Columbia University Irving Medical Center, New York, NY USA; 9 Department of Pathology and Cell Biology, Columbia University Irving Medical Center, New York, NY USA; 10 Department of Pathology, Yale School of Medicine, New Haven, CT USA; 11 Yale Cancer Center, Yale School of Medicine, New Haven, CT USA; 12 Yale Stem Cell Center, Yale School of Medicine, New Haven, CT USA; 13 Yale Center for Research on Aging (Y-Age), Yale School of Medicine, New Haven, CT USA; 14 Department of Statistics and Data Science, Yale University, New Haven, CT USA

**Keywords:** Cellular imaging, Optical imaging, Systems biology, Metabolism

## Abstract

Metabolism is fundamental to cell function, yet its activities vary across tissue environments. Resolving these processes in situ at single-cell resolution is crucial for understanding physiology in health and disease. However, existing methods lack biochemical specificity or direct linkage to cell identity. Here we report a method, Raman Enhanced Delineation of Cell Atlases in Tissues (REDCAT), an all-optical platform integrating Raman scattering microscopy and high-plex immunofluorescence to co-map metabolism and cell types. REDCAT achieves subcellular profiling of protein, lipid, nuclear metabolites and redox metabolism in human tissues. In lymph nodes, it revealed cell-type-specific metabolic specialization. In lymphoma, REDCAT uncovered profound reprogramming and transitional states during tumor transformation. In the liver, it resolved zonation-dependent metabolic gradients. By linking cell identity to spatial metabolic states, REDCAT provides a framework for studying immunity and cancer, offering a path to deciphering the metabolic basis of disease.

## Abstract (web summary)

Raman Enhanced Delineation of Cell Atlases in Tissues (REDCAT) integrates stimulated Raman scattering microscopy and high-plex immunofluorescence to profile single-cell proteins, lipids, nuclear metabolites and redox metabolism in tissue.

## Main

Metabolism influences cellular function in health and disease, including immunity and cancer^1^. It comprises the reactions that generate energy, synthesize biomolecules and maintain redox balance^2,3^, and is dynamically shaped by intrinsic programs and microenvironmental cues^3,4^. Measuring metabolism in situ at single-cell resolution is therefore essential. Although spatial omics can infer metabolic programs from transcripts or proteins^5–10^, these approaches do not directly report the dynamic metabolic state or robustly link metabolic activity to specific cell types and states.

Spatial metabolomics aims to capture tissue metabolic fingerprints across physiological states^11^. Mass spectrometry imaging (MSI) methods such as MALDI, DESI and SIMS can map spatial molecular distributions^12–15^ and, when combined with multiplex protein markers, profile metabolic heterogeneity with high sensitivity^16^. However, most MSI approaches are destructive and often trade molecular coverage for spatial resolution, limiting longitudinal studies and multimodal measurements on the same section. These constraints complicate integrated analyses and reduce the fidelity of linking metabolic measurements to cell identity at cellular or subcellular scales. On the other hand, Raman imaging enables label-free chemical mapping in aqueous biological samples and can resolve subcellular biomolecular composition and metabolite distributions^17–22^. Its inherent weak spontaneous signal is enhanced by stimulated Raman scattering (SRS), which provides a background-free, concentration-proportional contrast for quantitative imaging^23–25^. Hyperspectral SRS further yields Raman-like spectra for molecular discrimination and metabolic fingerprinting^26,27^, enabling integration with complementary modalities.

We developed REDCAT, a multimodal platform that integrates SRS metabolomic imaging, two-photon excited fluorescence (TPEF), second harmonic generation (SHG) and highly multiplexed immunofluorescence cell typing via CODEX. A computational pipeline based on MaxFuse^28^ aligns modalities and links single-cell protein markers to metabolic features, enabling submicron-resolution measurements of proteins, lipids, nuclear features and redox state. REDCAT is compatible with both fresh-frozen and formalin-fixed paraffin-embedded (FFPE) clinical specimens. Using REDCAT, we profiled normal and malignant human lymphoid tissues and normal human liver, revealing cell-type-specific metabolic programs, tumor-associated metabolic reprogramming and zonation-dependent gradients from the central vein to the portal vein.

## Results

### Design and workflow of REDCAT

**Fig. 1:**
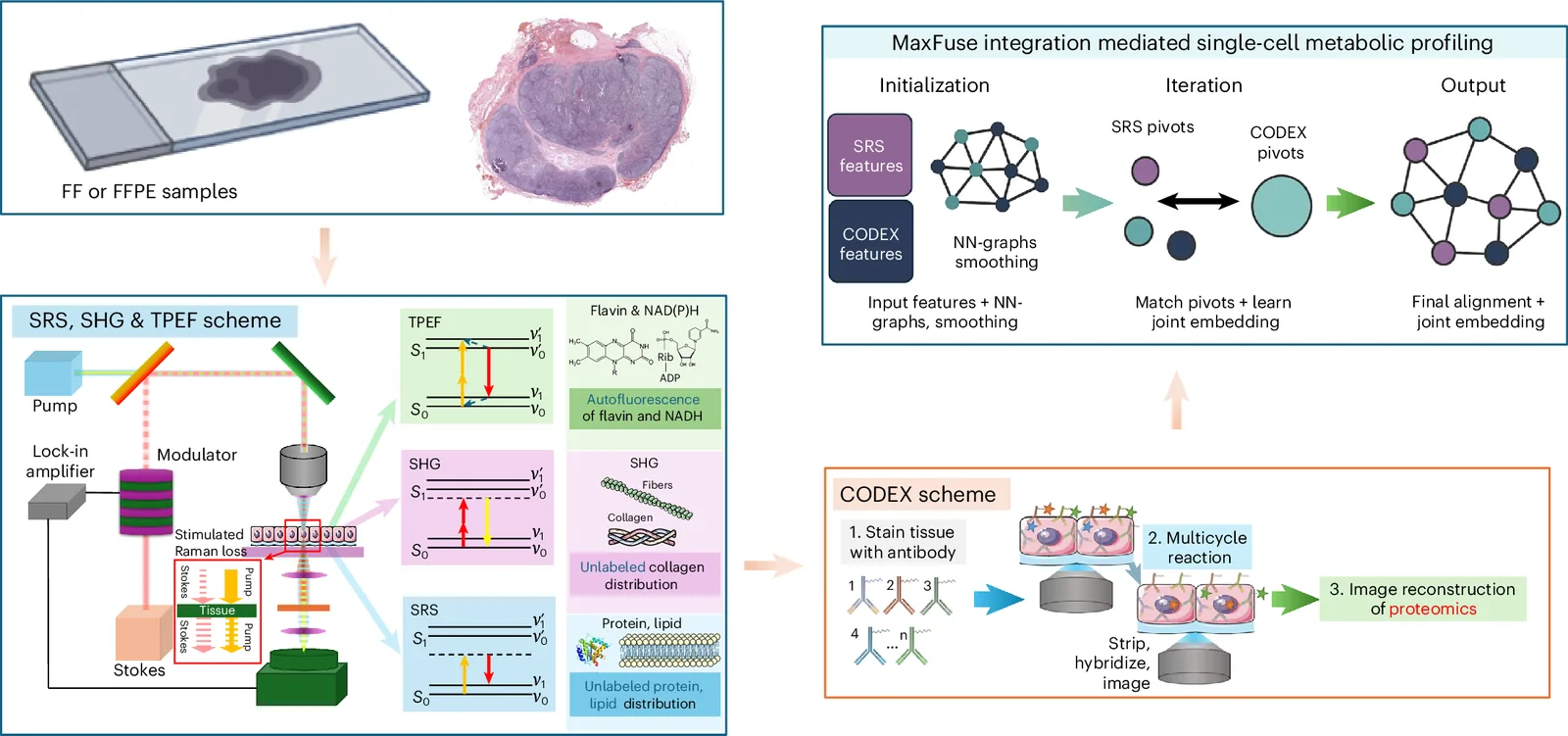
Design and workflow of REDCAT for integrated metabolic and proteomic profiling on the same tissue section. Schematic workflow. Fresh-frozen (FF) or FFPE tissue samples are sectioned and placed on a standard glass slide to firstly perform multimodal optical metabolic imaging that combines SRS, SHG and TPEF, and the same tissue section is subsequently imaged by highly multiplexed immunofluorescence such as CODEX for spatial protein profiling. Optical metabolic imaging includes autofluorescence for NADH and flavin (redox imaging), SHG for unlabeled collagen distribution and SRS for label-free protein and lipid mapping. CODEX multiplexed immunofluorescence workflow involves iterative antibody staining, stripping and imaging, followed by image reconstruction. It routinely images ~50 protein markers for precise cell typing of highly heterogeneous cell populations in tissue. The resulting metabolic maps of NADH/FAD ratio, lipid/protein ratio and collagen distribution as well as SRS hyperspectral imaging coupled with CODEX images from the same tissue section were fed into the MaxFuse pipeline. It created a nearest-neighbor (NN) graph for each modality, followed by the smoothing and matching process. Finally, co-registration of SRS and CODEX data enables the mapping of single-cell-resolved and cell-type-specific metabolic profiles.

The REDCAT workflow is summarized in Fig. 1. A ~10-µm FFPE or fresh-frozen section on glass is first imaged by the TPEF–SRS-SHG platform to capture label-free metabolic features at single-cell resolution. TPEF measures autofluorescence from reduced NAD(P)H and oxidized flavins (FAD/FMN) to compute an optical redox ratio (NADH/FAD), a proxy for cellular redox balance^29^; higher values indicate a more reduced state, consistent with increased glycolysis and/or impaired oxidative phosphorylation^30^. SRS acquires protein and lipid signals by tuning the pump wavelength to 2,930 cm^−1^, 2,850 cm^−1^, 3,015 cm^−1^ and 2,880 cm^−1^, respectively, enabling quantifying lipid/protein and unsaturated/saturated lipid ratios. SHG provides label-free collagen mapping. For detailed chemical profiling, hyperspectral SRS is collected across 2700–3,150 cm^−1^ (51 channels, ~10 cm^−1^ resolution). The same section is then profiled by ~50-plex CODEX immunofluorescence, followed by hematoxylin and eosin (H&E) staining. Images are aligned by affine registration, single-cell features are extracted after segmentation (Mesmer), and cells are matched across modalities with MaxFuse to generate joint embeddings that link protein-defined cell types to metabolic features in a cell-by-cell manner.

### REDCAT maps cell-type-specific metabolic activities in healthy human lymph nodes

**Extended Data Fig. 1:**
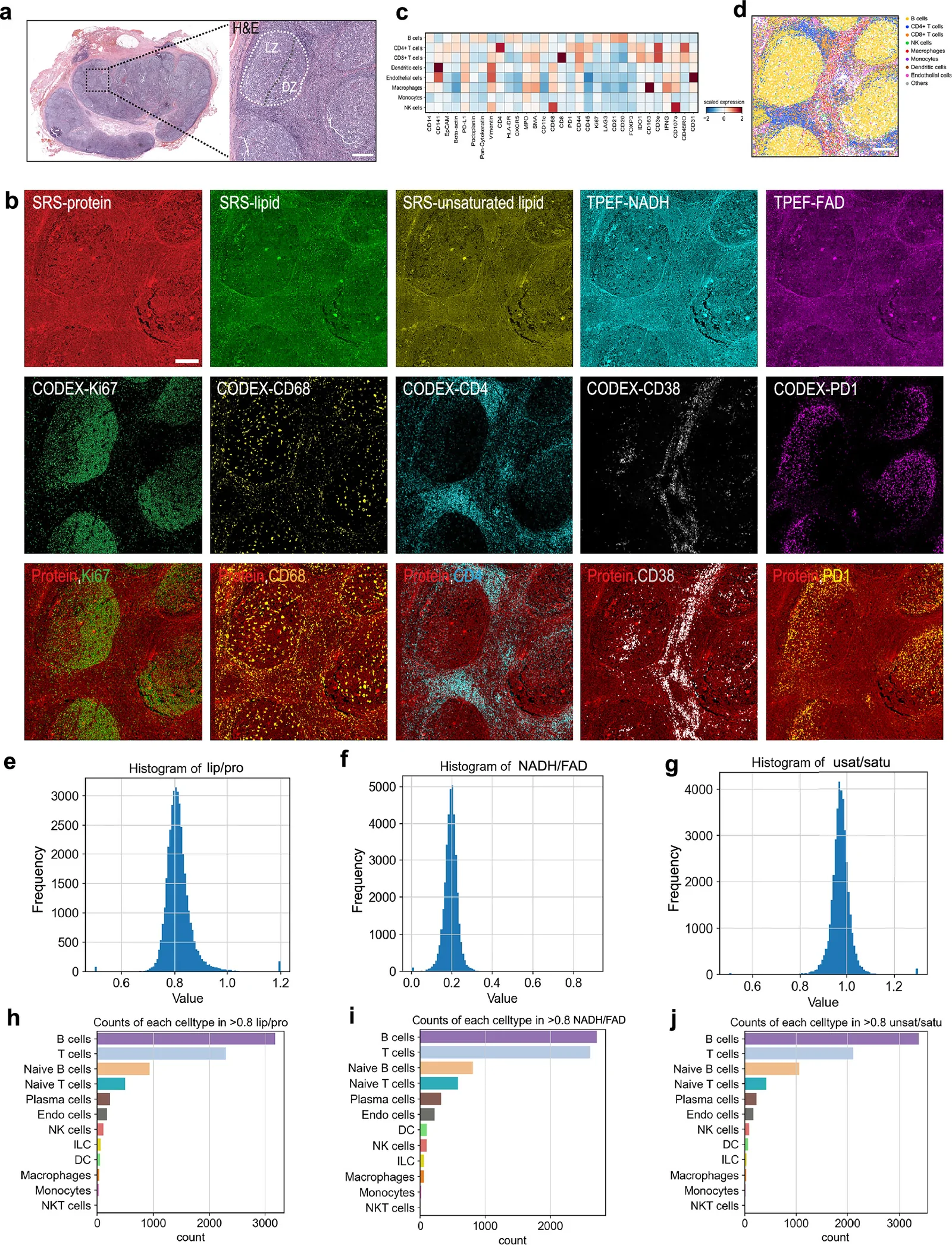
Multimodal metabolic and proteomic imaging of lymphoid tissue using REDCAT. **(a)** H&E-stained whole-section and magnified views showing the tissue architecture of normal lymph node. The geminal center (GC) light zone (LZ) and dark zone (DZ) were highlighted. Scale bar, 200 μm. **(b)** Multichannel imaging of the same tissue section: SRS-protein, SRS-lipid, SRS-unsaturated lipid, TPEF-NADH, and TPE- FAD autofluorescence; CODEX immunofluorescence for Ki67, CD68, CD4, CD38, and PD-1; and corresponding merged overlays of SRS protein with each immunostaining channel. Scale bar, 200 μm. **(c)** Heatmap showing scaled expression of selected protein markers across annotated immune cell types in normal lymph node. **(d)** Spatial mapping of annotated immune cell types based on protein expression in normal lymph node. Scale bar, 200 μm. **(e–g)** Histograms of lipid/protein ratio (**e**), NADH/FAD ratio (**f**), and unsaturated/saturated lipid ratio (**g**) for all single cells in normal lymph node. **(h–j)** Counts of each annotated cell type exceeding thresholds of lipid/protein ratio ≥0.8 quartile (**h**), NADH/FAD ratio ≥0.8 quartile (**i**), and unsaturated/saturated lipid ratio ≥0.8 quartile 8 (**j**), highlighting cell populations with metabolic ratios in the top quantile.

To demonstrate REDCAT for decoding normal immune physiology, we applied it to a deparaffinized FFPE lymph node tissue section from a healthy donor across a 1.6 mm × 1.6 mm region of interest (ROI; Extended Data Fig. 1a). Lymph nodes, as the central component of the secondary lymphoid system, are primarily composed of B and T lymphocytes, organized in coordination with dendritic cells, macrophages and stromal cells around spatially defined lymphoid follicles. Immune cells within these tissues undergo dynamic processes such as proliferation, differentiation, activation and responses to extracellular signals or stressors. These processes are tightly regulated by coordinated cell remodeling, niches and interactions accompanied by distinct metabolic reprogramming^31,32^.

**Fig. 2:**
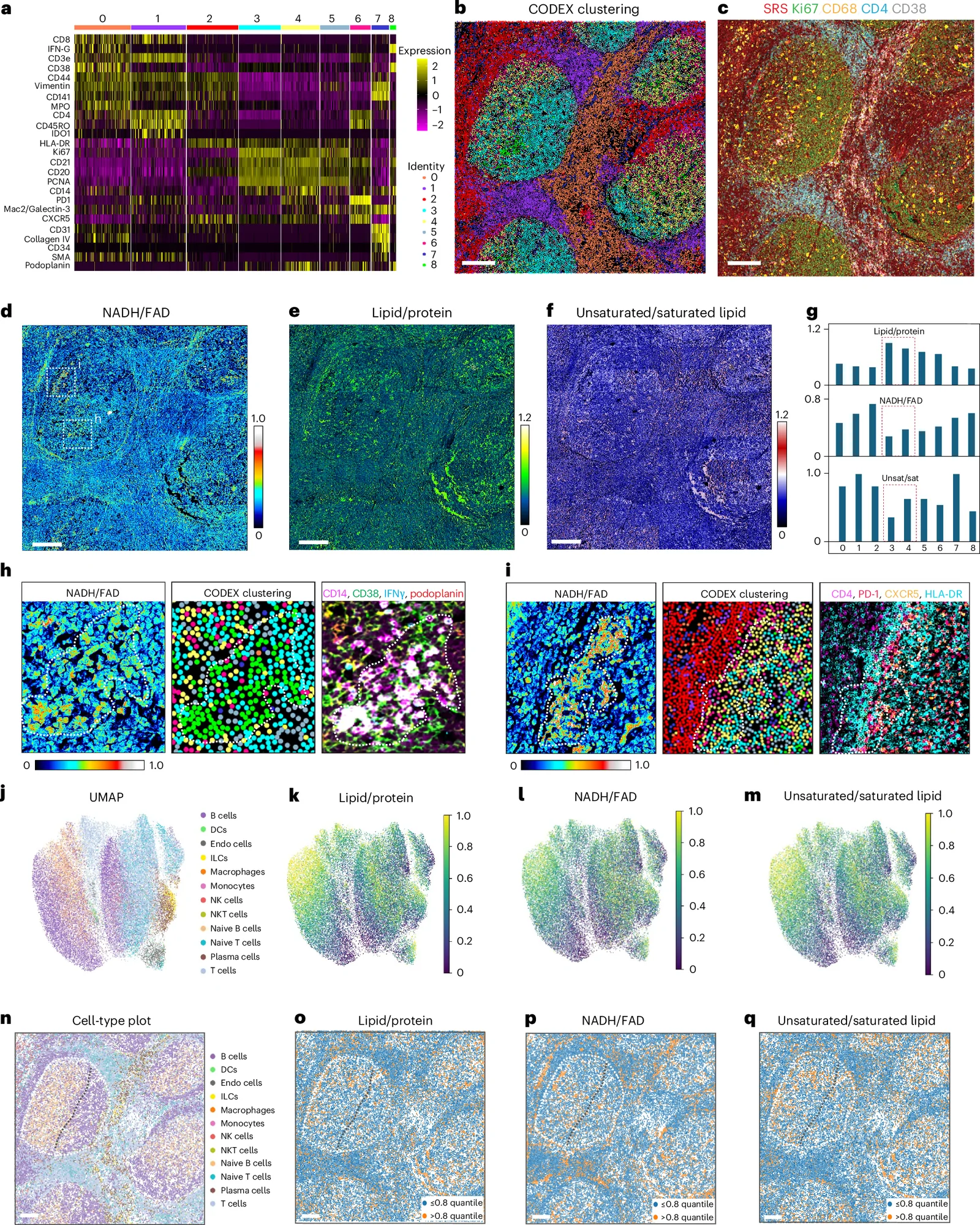
Integrated metabolic and cell-type profiling of human lymph node tissue via REDCAT. **a**, Heat map showing expression levels of protein markers across distinct cell clusters identified by CODEX imaging. Marker expression calibration bar (magenta–yellow) and cluster identities (color dots) are indicated. **b**, Spatial map of all nine cell clusters from unsupervised clustering of CODEX data after segmentation; cluster identities (color dots) are same as those shown in **a**. Scale bar, 200 μm. **c**, Representative CODEX image showing expression of Ki67 (green), CD68 (yellow), CD4 (cyan) and CD38 (white), which is further overlaid with the SRS signal (red) of CH_3_ vibrational modes (total protein signal). Scale bar, 200 μm. **d**–**f**, SRS-based metabolic maps of the same tissue section showing NADH/FAD optical redox ratio (**d**), lipid/protein ratio (**e**) and unsaturated/saturated lipid ratio (**f**). Scale bars, 200 μm. **g**, Quantification of lipid/protein, NADH/FAD and unsaturated/saturated lipid ratios across the identified cell clusters. Among them, cluster 3 (cyan in **b**) and cluster 4 (yellow in **b**) were highlighted. **h**, Enlarged view of the boxed region in **d** highlighting high NADH/FAD signal (left), corresponding CODEX cluster identities (middle, shown the same color dots as those shown in **a**; green color is the cluster 8 shown in **a** and immunofluorescence markers for CD14 (magenta), CD38 (green), IFNγ (cyan) and podoplanin (red; right). **i**, Enlarged view of the boxed region in **d** showing high NADH/FAD in a T_FH_ cell-rich area (left), corresponding CODEX clustering (middle, shown the same color dots as those shown in **a**) and immunofluorescence for CD4 (magenta), PD-1 (red), CXCR5 (fellow) and HLA-DR (cyan; right). **j**, UMAP visualization of single-cell clustering annotated by cell type. **k**–**m**, UMAP feature plots showing single-cell lipid/protein ratio (**k**), NADH/FAD (**l**) and unsaturated/saturated lipid ratio (**m**) generated by co-registration of multimodal SRS imaging with CODEX cell typing data. **n**, Spatial matrix plot displaying cell-type distribution across tissue architecture. Scale bar, 200 μm. **o**–**q**, Spatial maps of lipid/protein (**o**), NADH/FAD (**p**) and unsaturated/saturated lipid (**q**) ratios overlaid with cell-type annotations, highlighting metabolic heterogeneity in a follicular zone. DCs, dendritic cells. ILCs, innate lymphoid cells. Scale bars, 200 μm. Source data

**Extended Data Fig. 2:**
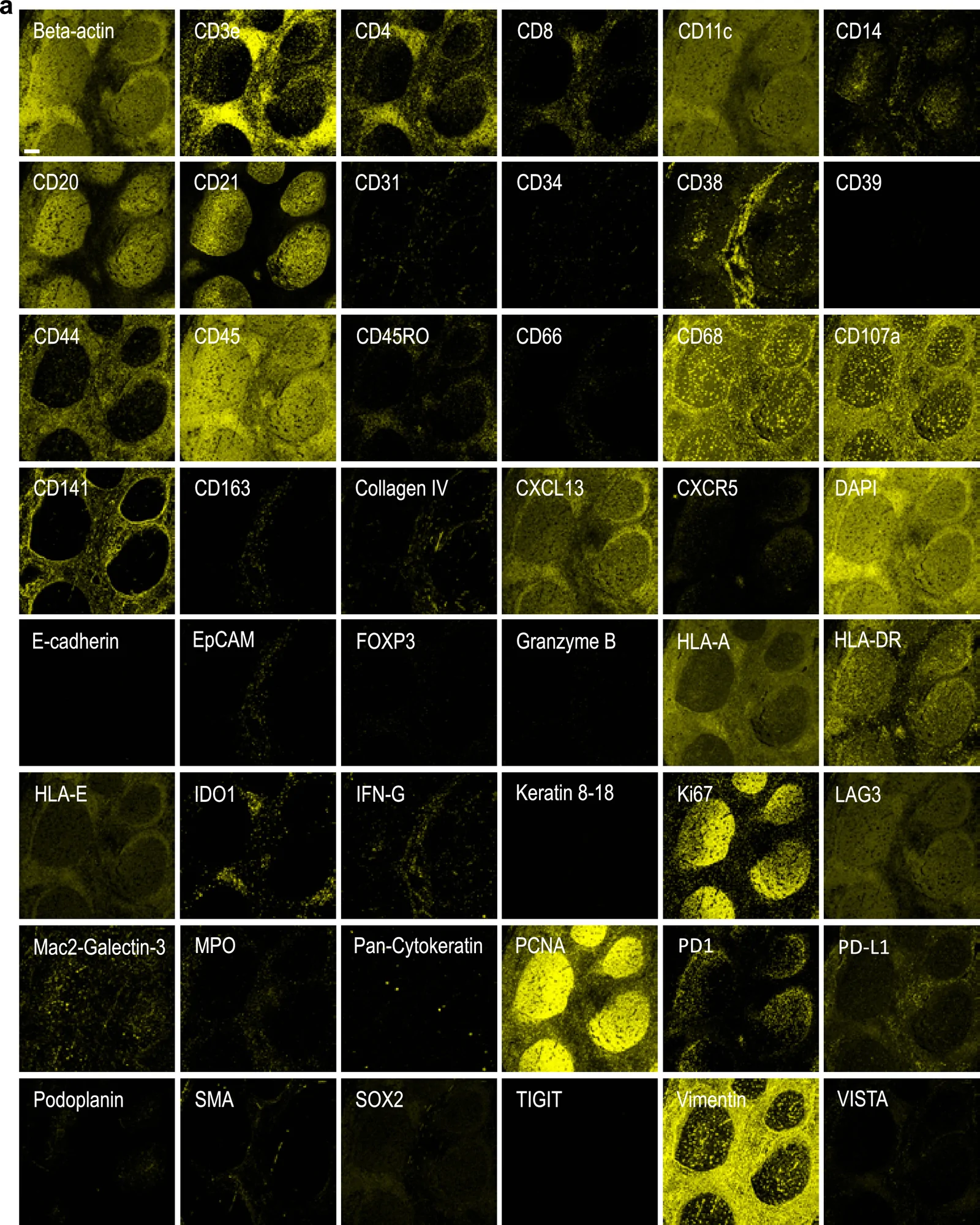
CODEX antibody panel validation in lymph node tissue sections. **(a)** Representative single-channel images from the 48-plex CODEX antibody panel showing specific staining patterns for immune, stromal, epithelial, and functional markers. Each panel corresponds to one antibody target (label indicated) acquired from the same tissue section. Markers include structural proteins (for example, Beta-actin, Vimentin), immune lineage markers (for example, CD3e, CD4, CD8, CD11c, CD14, CD20, CD21, CD38, CD45, CD68, CXCR5), stromal and extracellular matrix markers (for example, Collagen IV, SMA, Podoplanin), checkpoint/inhibitory molecules (for example, PD-1, PD-L1, TIGIT, LAG3, VISTA), and functional molecules (for example, Ki67, IFN-γ, Granzyme B, IDO1). DAPI channel shows nuclear counterstaining. All images were acquired under identical imaging conditions to ensure comparability of staining patterns. Scale bar, 200 μm.

To spatially investigate single-cell-type-specific metabolic and molecular features in this immune organ, NADH, FAD, protein and lipid signals were acquired using TPEF–SRS imaging (Extended Data Fig. 1b), followed by CODEX imaging on the same tissue section using an antibody panel of 48 protein markers (Extended Data Fig. 2a). This panel included surface markers for immune cells, cytokine receptors, proliferation markers and extracellular matrix proteins of interest. After cell segmentation and unsupervised clustering of single-cell protein expression profiles, nine distinct cell clusters were identified and spatially organized with known histological structures (Fig. 2a,b and Extended Data Fig. 1a). Clusters 3–6 and 8 corresponded to the follicular zone, and clusters 0–2 and 7 were associated with the interfollicular zone. After image integration, the SRS and CODEX images were co-registered for single-cell-level spatial correspondence (Fig. 2c and Extended Data Fig. 1b). TPEF–SRS imaging-based NADH/FAD, lipid/protein and unsaturated/saturated lipid ratiometric images were generated (Fig. 2d–f), and the ratio value of each CODEX cluster was extracted. Quantification analysis revealed a positive correlation between the unsaturated/saturated lipid ratio and the NADH/FAD ratio, but a negative correlation between the lipid/protein ratio and either the NADH/FAD or the unsaturated/saturated lipid ratio across most clusters (Fig. 2g). These relationships suggest that elevated lipid metabolic activity, particularly through lipid desaturation, is associated with a more reduced cellular redox state. In contrast, high lipid/protein ratios indicate an oxidative redox state. Cells in cluster 4 (yellow in Fig. 2a,b) in the germinal center light zone showed a higher NADH/FAD redox ratio and unsaturated-to-saturated lipid ratio than those in cluster 3 (cyan in Fig. 2a,b) in the dark zone (Extended Data Fig. 1a; H&E image). Both clusters strongly expressed CD20 and CD21, identifying them as germinal center B cells (Fig. 2a). This aligns with reports that germinal center dark-zone B cells (cluster 3) focus on rapid proliferation and genetic diversification, requiring high energy and biosynthetic flux, leading to oxidative stress and an oxidized redox state. In contrast, light zone (cluster 4) B cells are metabolically quiescent, engaging in antigen processing and presentation. They may enhance unsaturated lipid remodeling and redox buffering, supporting interactions with follicular helper T (T_FH_) cells. Additionally, cells in cluster 8 (green in Fig. 2a,b,h) between the germinal center light and dark zones showed a high NADH/FAD ratio (Fig. 2h). These cells expressed CD14, CD38, interferon gamma (IFNγ) and podoplanin, suggesting they are macrophages/monocytes or dendritic cells producing cytokines/chemokines in the germinal center. Their highly reduced redox state indicates active glycolysis (Fig. 2h). On the germinal center follicle’s apical side, T_FH_ cells expressing CD4, PD-1, CXCR5 and HLA-DR (Fig. 2i) showed a high NADH/FAD ratio. This glycolysis-associated metabolic phenotype likely reflects the energetic and biosynthetic demands of supporting B cell activation and affinity maturation during the germinal center reaction.

To evaluate the metabolic activity, we annotated the cells using protein expression profiles from the CODEX assay (Extended Data Fig. 1c). The high-plex protein panel clustered tissue pixels into distinct regions, classifying major immune and stromal cell types (Extended Data Fig. 1c,d), but lacked the resolution to distinguish finer immune cell subclasses. Existing markers cannot reliably differentiate naive, memory, plasma and germinal center B cells or specialized T cell subsets. To address this, we integrated CODEX data with a single-cell RNA-sequencing (scRNA-seq) atlas of lymph node cells using MaxFuse (Methods). This multimodal approach enhances subclass-level cell identity and functional annotation, aiding the detailed interpretation of cell-type-specific metabolic features. Using uniform manifold approximation and projection (UMAP) visualization, we clustered and annotated the cells into major immune and stromal populations, including B cells, T cells, dendritic cells, monocytes, macrophages, natural killer (NK) cells, endothelial cells and innate lymphoid cells (Fig. 2j).

We next extracted single-cell lipid/protein ratios, NADH/FAD redox ratios and unsaturated/saturated lipid ratios quantified from TPEF–SRS imaging and plotted their distributions as histograms (Extended Data Fig. 1e–g). Cells with ratio values in the top quantile (≥0.8 quartile) were predominantly B and T cells, consistent with their abundance in the lymph node (Extended Data Fig. 1h–j). To explore the metabolic state and cell-identity relationships, we overlaid these features onto the cell-type UMAP. The results showed a broad metabolic state distribution within each cell-type cluster, including B cells, T cells, monocytes and macrophages (Fig. 2k–m). This suggests that cell identity alone does not fully define metabolic phenotypes, indicating that context-dependent states, such as activation, differentiation or spatial localization within tissues, may influence metabolic reprogramming.

To resolve single-cell metabolic phenotypes across cell types, we reprojected the cell types identified by MaxFuse integration of CODEX and snRNA-seq data into the original tissue coordinates using matrix plotting (Fig. 2n). The metabolic features (NADH/FAD, lipid/protein and unsaturated/saturated lipid ratios) did not uniformly match specific cell types (Fig. 2o–q). However, spatial patterns have emerged. Within the germinal center, most B cells in the light zone showed higher NADH/FAD ratios and lipid desaturation than those in the dark zone (Fig. 2n,p,q). Although these features are not always coexpressed within individual B cells owing to metabolic heterogeneity, their regional enrichment highlights distinct metabolic programs within the germinal center microenvironment, even within the same cell type. These findings corroborate the CODEX cluster analysis and suggest that B cells in the light zone exhibit a more reduced redox state and anabolic lipid metabolism linked to B cell selection and T cell help, whereas dark-zone B cells show lower NADH/FAD and unsaturated lipid ratios, consistent with a more oxidized redox state characteristic of high proliferation and somatic hypermutation. These findings demonstrate the effectiveness and accuracy of the REDCAT pipeline in mapping single-cell metabolic activity and tissue-level metabolic heterogeneity.

### REDCAT reveals metabolic reprogramming in human lymphoma

**Extended Data Fig. 3:**
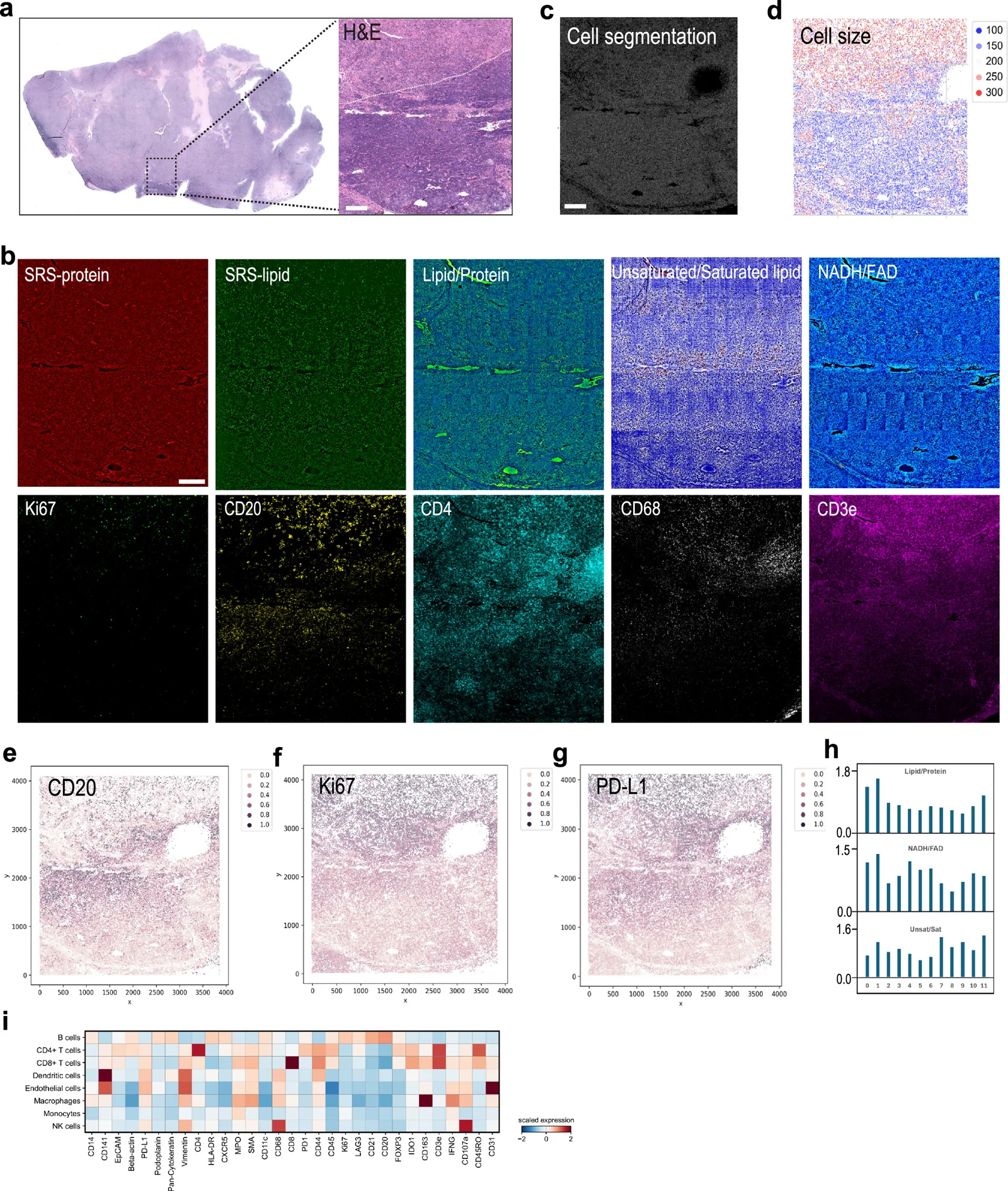
Multimodal metabolic and proteomic imaging of lymphoma tissue. **(a)** Whole-section and magnified H&E images of lymphoma ROI showing tumor architecture. Scale bar, 200 μm. **(b)** Multimodal imaging including SRS-protein, SRS-lipid, lipid/protein ratio, unsaturated/saturated lipid ratio, NADH/FAD ratio, and CODEX immunofluorescence for Ki67, CD20, CD4, CD68, and CD3e. Scale bar, 200 μm. **(c)** Cell segmentation of the same region shown in (**a, b**) to quantify the cell size. Scale bar, 200 μm. **(d)** Spatial map of cell size distribution across the lymphoma ROI shown in (**a, b**). **(e–g)** Whole-slide spatial distribution maps of CD20 (**e**), Ki67 (**f**), and PD-L1 (**g**) expression derived from CODEX imaging. **(h)** Quantification of lipid/protein ratio, NADH/FAD ratio, and unsaturated/saturated lipid ratio across CODEX identified cell clusters of the lymphoma ROI. **(i)** Heatmap of marker expression across annotated cell types in the lymphoma ROI.

Cancer cells in rapid proliferation must alter their metabolism to meet increased energy and biosynthesis demands. This reprogramming depletes nutrients, creating a hypoxic and acidic tumor microenvironment, impairing antitumor immune cell activation and recruitment^2,31^. We used REDCAT to assess cancer and immune cell metabolic activities in a lymphoma tissue sample, aiming to identify novel cell-type-specific metabolic processes for therapeutic strategies. The sample included chronic lymphocytic leukemia (CLL), a low-grade lymphoma, adjacent to diffuse large B cell lymphoma (DLBCL), an aggressive subtype. This rare clinical phenomenon, captured in the same biopsy, is known as Richter’s transformation (Extended Data Fig. 3a)^33^. The H&E image (1.8 mm × 2.0 mm) showed small, monomorphic tumor cells with dense packing, transitioning to a loosely packed area with pleomorphic large tumor lymphocytes in the upper region (Extended Data Fig. 3a).

**Fig. 3:**
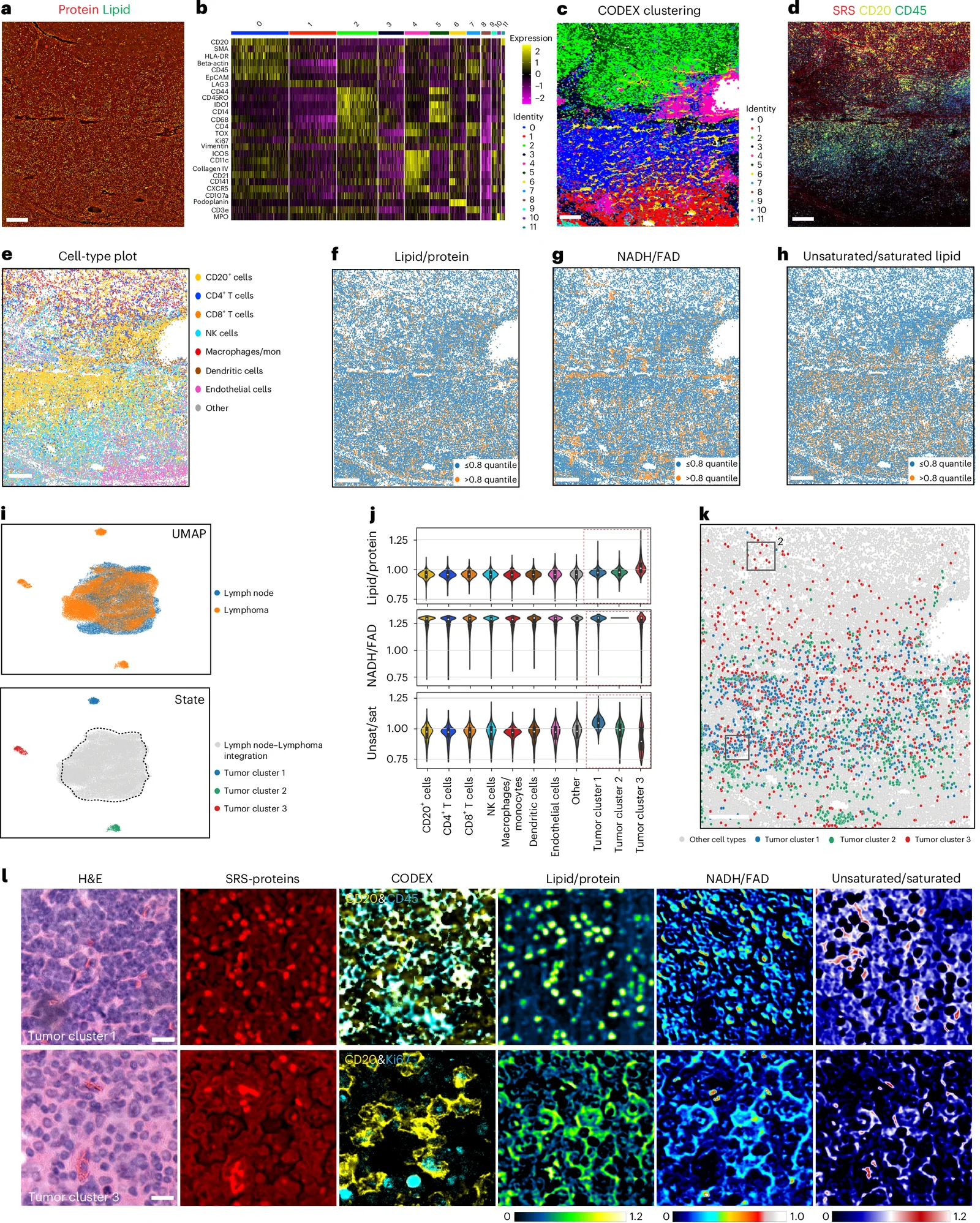
Spatial metabolic and phenotypic profiling of lymphoma using REDCAT. **a**, SRS image showing protein (red) and lipid (green) distribution in a lymphoma tissue section. Scale bar, 200 μm. **b**, Heat map of 35-marker expression profiles across 12 distinct cell clusters identified by CODEX imaging, with cluster identities indicated by the color bar. **c**, Spatial representation of CODEX unsupervised clustering results in lymphoma tissue. Scale bar, 200 μm. **d**, Overlay of SRS metabolic imaging with CODEX immunofluorescence for CD20 (yellow), CD3 (cyan) and CD68 (red), showing spatial relationships between metabolic features and immune markers in lymphoma tissue. Scale bar, 200 μm. **e**–**h**, Co-registered spatial maps of immune cell types (matrix plot; **e**), lipid/protein ratio (**f**), NADH/FAD ratio (**g**) and unsaturated/saturated lipid ratio (**h**), highlighting metabolic heterogeneity across cell populations in lymphoma tissue. Scale bars, 200 μm. **i**, UMAP integrating cell annotations from lymphoma and normal lymph nodes identified three distinct tumor cell clusters distinguished by their protein expression profiles (top). These three distinct tumor cell clusters have different metabolic states (bottom). **j**, Violin plots comparing lipid/protein ratio, NADH/FAD ratio and unsaturated/saturated lipid ratio among different cell types and tumor clusters in lymphoma tissue. The metabolic values were shown as mean ± s.e.m. Each value represents the individual cell, and each violin plot represents the total cell count from each annotated cell type. **k**, Spatial distribution of cells assigned to tumor cluster 1 (blue), tumor cluster 2 (green), tumor cluster 3 (red) and other cell types (gray). **l**, Representative histological and multimodal imaging features of tumor cluster 1 (top; ROI 1 outlined in **k**) and tumor cluster 3 (bottom; ROI 2 outlined in **k**), including H&E staining, SRS protein imaging, CODEX immunofluorescence (CD45 or Ki67 and CD20), lipid/protein ratio, NADH/FAD ratio and unsaturated/saturated lipid ratio maps. Scale bars, 20 μm. Source data

**Extended Data Fig. 4:**
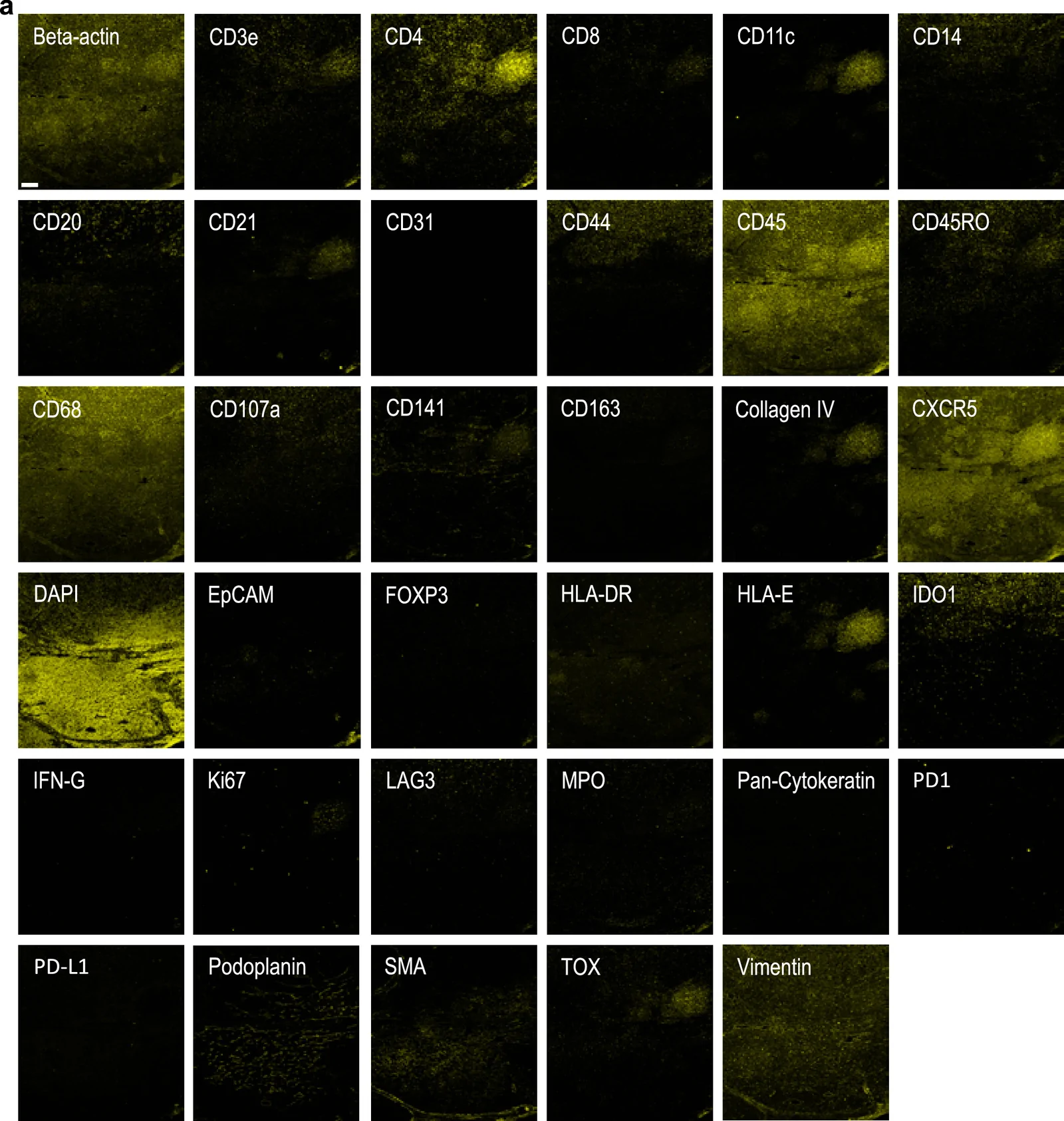
CODEX antibody panel validation in tissue sections. **(a)** Representative single-channel images from the CODEX antibody panel showing staining patterns for structural, immune, stromal, and functional markers. Markers include Beta-actin, immune lineage markers (for example, CD3e, CD4, CD8, CD11c, CD14, CD20, CD21, CD44, CD45, CD45RO, CD68, CD107a, CD141, CD163, CXCR5), stromal/extracellular matrix proteins (for example, Collagen IV, SMA, Podoplanin, Vimentin), checkpoint/inhibitory molecules (for example, PD-1, PD-L1, LAG3, TOX, IDO1), and functional markers (for example, Ki67, FOXP3, IFN-γ, MPO). The DAPI channel is included for nuclear counterstaining. All images were acquired under identical imaging conditions for direct comparison of staining specificity and intensity. Scale bar, 200 μm.

To study the metabolic activities in this lymphoma sample, we acquired NADH, FAD, protein and lipid signals, generating quantitative radiometric images via TPEF–SRS imaging (Fig. 3a and Extended Data Fig. 3b), followed by CODEX imaging for 35 protein markers in the same ROI (Extended Data Fig. 4a). The sample showed a high lipid content as scattered puncta with elevated intensity (Fig. 3a). Unsupervised clustering of CODEX data revealed 12 cell clusters, which were remapped to visualize cellular identities within the tissue (Fig. 3b,c). Cluster 11 mainly comprised DLBCL cells with high CD20, PD-L1 and Ki67 expression (Fig. 3b,c), located in the upper ROI (Fig. 3a and Extended Data Fig. 3b). Morphologically, DLBCL cells had large bodies with irregular nuclei and dense nucleoli, which were distinct from those of other immune cells (Fig. 3d). These features were validated by cell size quantification (Extended Data Fig. 3c,d) and the immunofluorescence intensity of CD20, PD-L1 and Ki67 after cell segmentation (Extended Data Fig. 3c,e–g), confirming the histopathological diagnosis. We analyzed NADH/FAD, lipid/protein and unsaturated/saturated lipid ratios across CODEX-defined clusters and found that clusters 0 and 1 (B cells, myeloid cells/macrophages and some T cells) had high lipid and redox ratios (Extended Data Fig. 3h), indicating elevated lipid biosynthesis. Other clusters showed no consistent trends across these parameters (Extended Data Fig. 3h), in contrast to the coordinated profiles in normal lymph nodes, highlighting the disrupted metabolic homeostasis in the tumor microenvironment.

**Extended Data Fig. 5:**
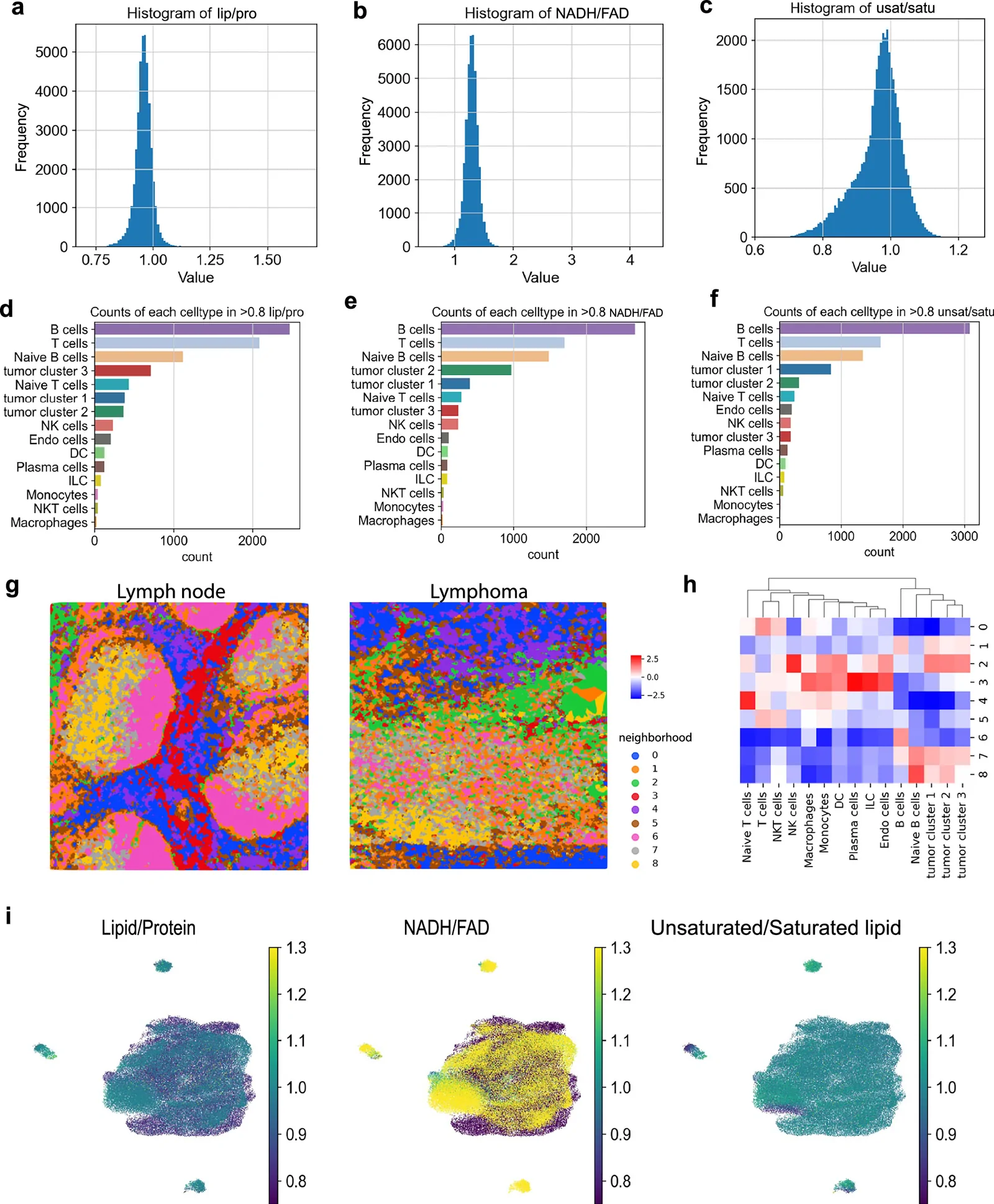
Single-cell metabolic profiling and neighborhood analysis of lymph node and lymphoma. **(a–c)** Histograms of lipid/protein (**a**), NADH/FAD (**b**), and unsaturated/saturated lipid (**c**) ratios for all single cells in lymphoma. **(d–f)** Counts of each annotated cell type with metabolic ratios greater than 0.8 for lipid/protein (**d**), NADH/FAD (**e**), and unsaturated/saturated lipid (**f**), highlighting metabolically distinct subsets in lymphoma. **(g)** Spatial neighborhood maps for lymph node (left) and lymphoma (right), showing cellular neighborhood composition. **(h)** Heatmap of neighborhood enrichment scores across cell types and tumor clusters, identifying microenvironmental differences between lymph node and lymphoma. **(i)** UMAP feature plots showing single-cell lipid/protein ratio, NADH/FAD ratio, and unsaturated/saturated lipid ratio in both lymph node and lymphoma.

To investigate the single-cell metabolic activity, we performed annotation (Extended Data Fig. 3i) and spatially located each individual cell in tissue (Fig. 3e). We quantified the metabolic features by plotting the histogram distributions of the metabolic ratios (Extended Data Fig. 5a–c). The unsaturated lipid ratios in lymphoma were broader than those in normal lymph nodes (Extended Data Figs. 1f and 5c), indicating greater unsaturation heterogeneity in lymphoma tissue, possibly reflecting tumor cells at different stages interacting with the surrounding cells. In lymphoma tissue, cells with top-quartile metabolic ratios (≥0.8 quartile) were mainly B and T lymphocytes (Extended Data Fig. 5d–f). However, unlike normal tissue, tumor cells also made up a substantial fraction of these top-quartile cells (Extended Data Fig. 5e–f), indicating widespread metabolic reprogramming within the tumor niche. When mapped to spatial coordinates, cells with high metabolic ratios were dispersed but concentrated in the lower ROI, where CLL cells predominated (Fig. 3f–h). The metabolic features of lymphoma lacked spatial correlation with the tissue architecture seen in normal lymph nodes, possibly due to the disordered structure of lymphoma tissues.

To compare the immune cell organization between normal and tumor tissues, we applied spatial neighborhood analysis to both. In normal lymph nodes, spatial neighborhoods showed clear compartmentalization, matching anatomical zones (Extended Data Fig. 5g,h). Neighborhood clusters rich in naive B cells, plasma cells and follicular dendritic cells (clusters 2, 3 and 5) were found in B cell follicles, while macrophage-enriched and dendritic cell-enriched clusters (clusters 0 and 7) were mainly in interfollicular and paracortical T cell zones, reflecting a coordinated immune architecture. Conversely, the lymphoma sample showed a disrupted spatial architecture, with intermixed and less compartmentalized neighborhoods (Extended Data Fig. 5g,h). Some B cell-rich clusters remained, but with less distinct boundaries than before. There was an expansion of macrophage-rich and NKT cell-rich neighborhoods (clusters 0 and 1) and fewer naive B cell-dominated clusters, indicating a shift in tissue composition. The accompanying heat map (Extended Data Fig. 5h) quantitatively captured the altered cell-type enrichment between normal and tumor tissues. These findings align with DLBCL being of the activated B cell subtype from post-germinal center B cells. Lymphoma-associated neighborhoods showed increased heterogeneity, with normal immune niches partially replaced by tumor-associated microenvironments. These data highlight the profound remodeling of immune cell spatial organization in lymphoma, with disrupted follicular structures, altered neighborhood composition and potential impacts on immune metabolism and function.

By integrating metabolic features and protein expression profiles from lymphoma and normal lymph nodes (Methods), UMAP analysis identified three distinct tumor cell clusters based on their protein expression profiles (Fig. 3i,j). Compared to normal lymph nodes, lymphoma tissues had elevated total lipids, unsaturated lipids and NADH/FAD redox ratio (Extended Data Fig. 5i), indicating enhanced lipid metabolism and redox activity in the tumor microenvironment. Among the cell types, tumor cluster 1 had a high unsaturated/saturated lipid ratio, whereas cluster 3 had a lower unsaturated lipid ratio but a higher lipid-to-protein ratio (Fig. 3i). Notably, cluster 3 displayed a heterogeneous unsaturated lipid ratio with a bimodal pattern in the bar plot (Fig. 3i), which was confirmed by the UMAP plot (Extended Data Fig. 5i). Cluster 2 appeared to be transitional between clusters 1 and 3, with intermediate metabolic values (Fig. 3i), potentially reflecting a shift from CLL to DLBCL. These findings suggest high metabolic heterogeneity among tumor subclusters.

To visualize the metabolic heterogeneity in tumor clusters, we reprojected the single-cell data onto tissue coordinates and found that tumor cluster 1 mainly contained CLL cells and cluster 3 mostly contained DLBCL cells (Fig. 3k). Zoomed-in images revealed that CLL cells exhibited prominent lipid accumulation (Fig. 3l). These excess lipids, which can be acquired from the microenvironment or synthesized de novo, are stored as large lipid clusters. In contrast, DLBCL cells displayed a more diffuse lipid signal in the cytoplasm (Fig. 3l), suggesting lipid metabolism adaptation for rapid biosynthesis and energy in large B cells. We also noted a higher NADH/FAD redox ratio in DLBCL cells than in nonmalignant cells (Fig. 3l), consistent with studies showing increased glucose uptake and glycolytic activity in DLBCL cells^33^. The metabolic activity shift between these tumor subtypes suggests that malignant B cells and their surroundings are metabolically reprogrammed during the transformation from indolent to aggressive lymphoma to adapt to hypoxia and nutrient deprivation.

Collectively, REDCAT allows spatial elucidation of distinct metabolic states in lymphoma tissues and suggests a transitional metabolic state subpopulation involved in CLL-to-DLBCL transformation, enhancing our understanding of metabolic reprogramming in heterogeneous tumor microenvironment cell types.

### REDCAT resolved the change of cell-type-specific chemical profiles during lymphoma transformation

**Fig. 4:**
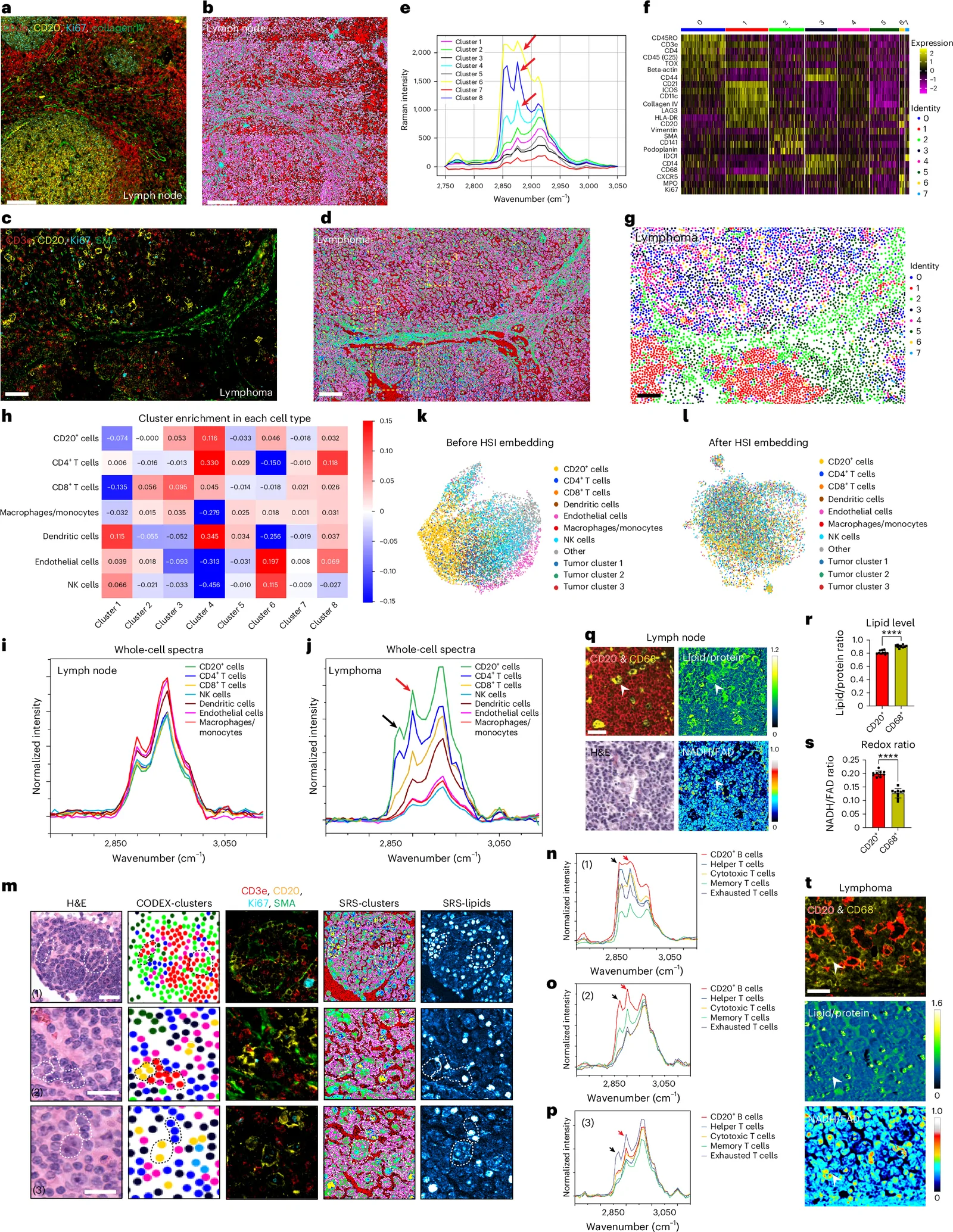
Comparison of metabolic and phenotypic states in normal lymph nodes and lymphoma. **a**, CODEX image of human lymph node tissue showing CD3e (red), CD20 (yellow), Ki67 (cyan) and αSMA (red), delineating the major cell types. Scale bar, 100 μm. **b**, SRS-based spatial *k*-means clustering map of lymph node tissue. Scale bar, 100 μm. **c**, CODEX image of a tissue section from transformation of CLL to DLBCL, stained for CD3e (red), CD20 (yellow), Ki67 (cyan) and αSMA (green). Scale bar, 100 μm. **d**, SRS *k*-means clustering map of the lymphoma sample, with highlighted regions corresponding to CLL (1), intermediate lymphoma (2) and DLBCL (3). Scale bar, 100 μm. **e** Integrated mean SRS spectra of *k*-means-defined clusters in normal lymph nodes and lymphoma. Red arrows indicate spectra from clusters 4, 6 and 8, which were enriched across multiple cell types in lymphoma. SRS spectra were processed by baseline subtraction and smoothing, followed by a two-step normalization strategy by preserving the intrinsic spectral shape and relative peak relationships within each spectrum as well as the inter-spectral intensity relationships. **f**, Heat map of marker expression across clusters in lymphoma, with identity assignments shown in the right color bar. **g**, CODEX spatial clustering map of lymphoma. Scale bar, 100 μm. **h**, Heat map showing enrichment of each immune cell type within *k*-means spectral clusters in lymphoma. **i**,**j**, Average SRS spectra of annotated immune cell types in lymph nodes (**i**) and lymphoma (**j**). In **j**, the red arrow indicates the peak at 2,880 cm^−1^, and the black arrow points to the peak at 2850 cm^−1^. SRS spectra were processed by baseline subtraction and smoothing, followed by a two-step normalization strategy by preserving the intrinsic spectral shape and relative peak relationships within each spectrum as well as the inter-spectral intensity relationships. **k**,**l**, UMAP plots of single-cell populations in lymphoma colored by cell type or tumor cluster before (**k**) and after (**l**) HSI embedding. **m**, Representative multimodal images of selected regions showing H&E, CODEX clusters, CODEX immunofluorescence (CD3e, CD20, Ki67, αSMA), SRS protein channel and SRS lipid channel (cyan hot; lipid signal was collected at 2,850 cm^−1^; the brighter the color, the greater the lipid enrichment). Zoomed-in views of the regions highlighted in **d**, corresponding to (1) CLL region, (2) intermediate region and (3) DLBCL region, are marked. Lipid accumulation is highlighted in these regions for the tumor cells and their surrounding microenvironment. Scale bars, 20 μm. **n**–**p**, SRS spectra of cell types from the indicated lymphoma regions (1), (2) and (3) shown in **m**. The red arrow indicates the peak at 2,880 cm^−1^; the black arrow highlights the peak at 2850 cm^−1^. SRS spectra were processed by baseline subtraction and smoothing, followed by a two-step normalization strategy by preserving the intrinsic spectral shape and relative peak relationships within each spectrum as well as the inter-spectral intensity relationships. **q**, Representative images in the normal lymph node tissue showing CD20 and CD68 immunostaining (top left), lipid/protein SRS ratio map (top right), H&E staining (bottom left, to show a typical macrophage containing lysosomes) and NADH/FAD redox map (bottom right). The white arrowhead indicates a CD68^+^ macrophage. Scale bar, 20 μm. **r**,**s**, Quantification of lipid/protein ratio (**r**) and NADH/FAD ratio (**s**) in CD68^+^ macrophages and CD20^+^ B cells in normal lymph node. Values are the mean ± s.e.m. ****P < 0.0001 (two-tailed Student’s *t*-test). *n* = 3 different ROIs. **t**, Representative images of the DLBCL region showing CD20 and CD68 immunostaining (top), lipid/protein SRS ratio (middle) and NADH/FAD redox (bottom). The white arrowhead indicates a CD68^+^ macrophage. Scale bar, 20 μm.

**Extended Data Fig. 6:**
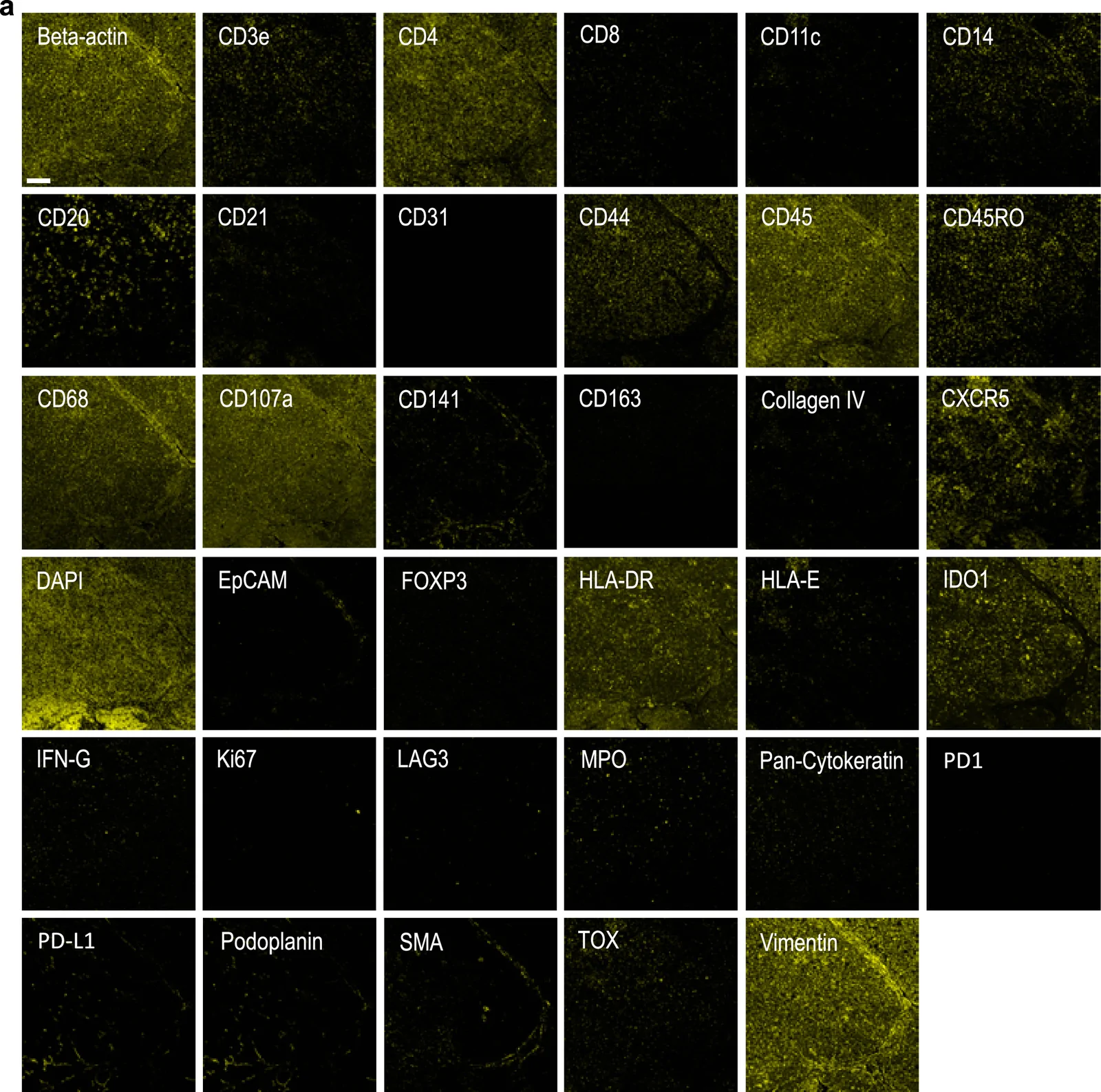
CODEX antibody panel validation in lymphoma tissue. **(a)** Representative single-channel images from the 35-plex CODEX antibody panel showing staining specificity for structural, immune, stromal, and functional markers. Markers include Beta-actin, immune lineage markers (for example, CD3e, CD4, CD8, CD11c, CD14, CD20, CD21, CD44, CD45, CD45RO, CD68, CD107a, CD141, CD163, CXCR5), stromal/extracellular matrix proteins (for example, Collagen IV, SMA, Podoplanin, Vimentin), checkpoint/inhibitory molecules (for example, PD-1, PD-L1, LAG3, TOX, IDO1), and functional/activation markers (for example, Ki67, FOXP3, IFN-γ, MPO). DAPI staining is included for nuclear visualization. All channels were imaged under identical acquisition conditions to ensure comparability of staining intensity and localization. Scale bar, 200 μm.

**Extended Data Fig. 7:**
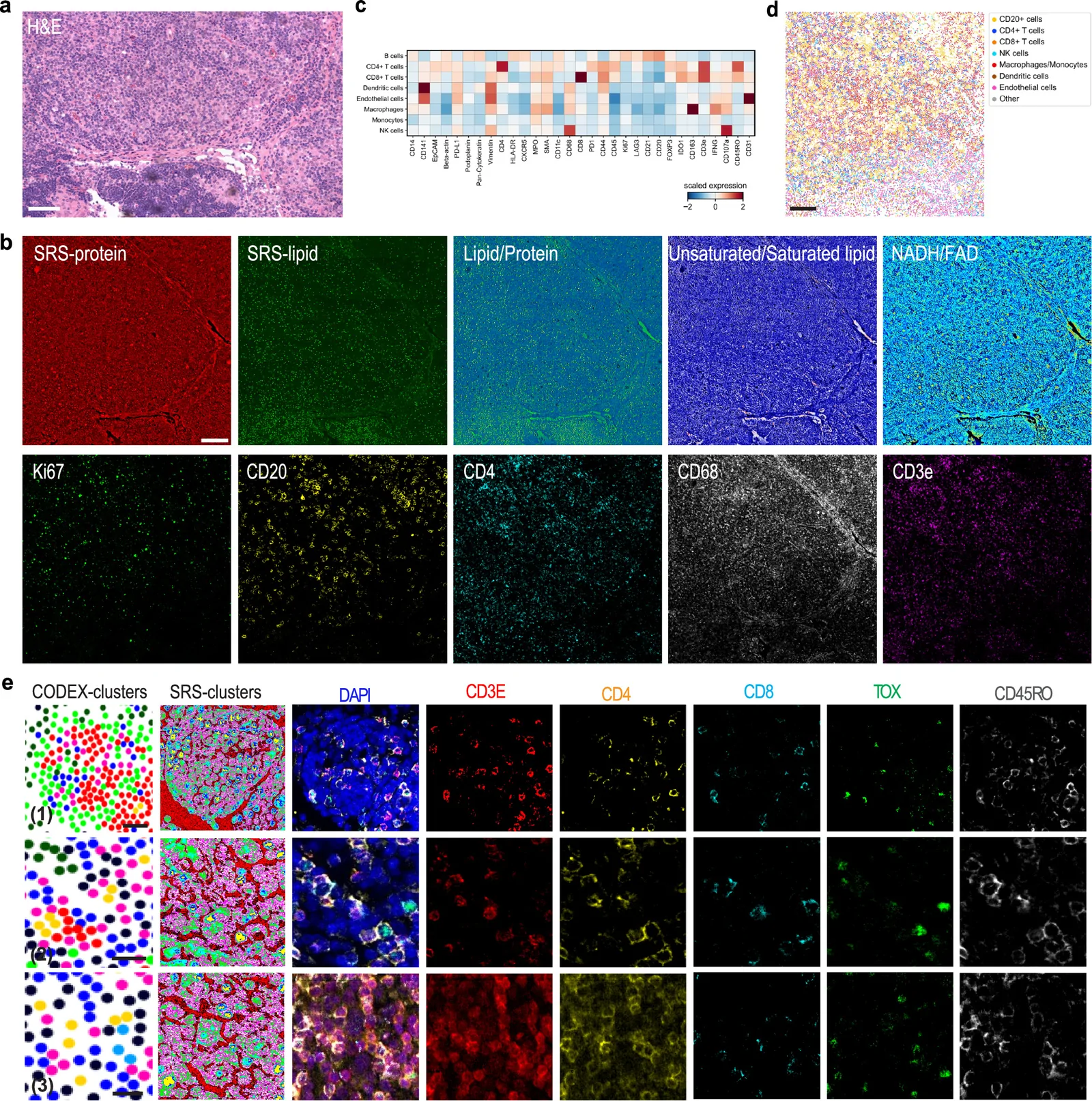
Multimodal metabolic and proteomic characterization of immune populations in lymphoma tissue. **(a)** H&E image of the lymphoma ROI containing low-grade and high-grade components in the lymphoma tissue, as well as a transformation interface between these two compartments. Scale bar, 200 μm. **(b)** Heatmap of scaled expression values for selected markers across annotated immune cell types, including CD4^+^ T cells, CD8^+^ T cells, CD20^+^ B cells, dendritic cells, endothelial cells, macrophages/monocytes, and NK cells in the lymphoma ROI. Scale bar, 200 μm. **(c)** Spatial map of annotated immune cell types across the lymphoma ROI. **(d)** Co-registered SRS and CODEX imaging of the same lymphoma ROI showing SRS-protein, SRS-lipid, lipid/protein ratio, unsaturated/saturated lipid ratio, NADH/FAD ratio, and CODEX immunofluorescence for Ki67, CD20, CD4, CD68, and CD3e. Scale bar, 200 μm. **(e)** Representative regions from three distinct microenvironments (Fig. 4m) showing CODEX cluster assignments, corresponding SRS cluster overlays, and high-magnification immunofluorescence for DAPI, CD3e, CD4, CD8, TOX, and CD45RO, illustrating the major T cell subtypes and spatial organization. Scale bars, 20 μm.

Hyperspectral SRS imaging (HSI) captures subcellular metabolic changes, with each image pixel containing a full Raman spectrum for chemical profiling at submicron resolution. We applied 35-panel CODEX and high-resolution SRS-HSI (0.25 µm per pixel, 10 cm^−1^ spectral resolution) to normal lymph nodes and the lymphoma ROI to analyze cell-type-specific metabolic profiles during CLL-to-DLBCL transformation (Fig. 4a–e and Extended Data Fig. 6a). Histological examination confirmed a clear boundary between low-grade and high-grade components, allowing precise spatial metabolic analysis (Extended Data Fig. 7a). Using unsupervised *k*-means clustering of SRS spectra, we identified eight chemical clusters (Fig. 4b,d,e). Tumor tissue showed enrichment of clusters 4, 6 and 8, with lipid-associated peaks (2,850 cm^−1^ and 2,880 cm^−1^), indicating increased lipid accumulation. This was corroborated by the SRS image at 2,850 cm^−1^ and lipid images (Extended Data Fig. 7b), showing enriched lipid signals. SRS-HSI clusters correlated spatially with CODEX clusters at the single-cell level (Fig. 4d,g). We integrated HSI results with CODEX-derived cell annotations (Extended Data Fig. 7c,d) and visualized spectral cluster enrichment by matrix plot, showing metabolic heterogeneity in tumor tissues. Clusters 4, 6 and 8, with lipid-associated spectra (Fig. 4c), were enriched across cell types, indicating widespread lipid remodeling. This aligns with SRS spectra showing spectral remodeling in tumor-infiltrating immune cells (Fig. 4i,j). Integration of CODEX-based protein expression with redox ratio, lipid-to-protein ratio and unsaturated-to-saturated lipid ratio revealed three tumor clusters besides major cell types in UMAP (Fig. 4k). The cell-type clustering pattern was disrupted after embedding hyperspectral data, highlighting metabolic heterogeneity within similar immune cell types (Fig. 4l). Three clusters emerged away from the main body, suggesting new cell networks (Fig. 4l).

To investigate cell–cell interactions in the tumor microenvironment and capture the metabolic trajectory from CLL to DLBCL, we defined CD20^+^ B cells between CLL and DLBCL regions as intermediate cells and compared their metabolic profiles with large B cells and CLL cells in the respective regions (Fig. 4m). CD20^+^ intermediate cells showed distinct SRS spectra from CLL and DLBCL cells (Fig. 4m–p), notably at lipid-associated peaks 2,850 cm^−1^ and 2,880 cm^−1^. This supports the existence of a putative intermediate state (tumor cluster 2) during the CLL-to-DLBCL transition. These findings support a stepwise metabolic reprogramming model, refining the shift from glycolysis to oxidative phosphorylation linked to the Richter transformation^34^.

Intriguingly, T cells in the three regions exhibited divergent chemical profiles (Fig. 4m–p and Extended Data Fig. 7e). These included CD4^+^CD3e^+^ helper T cells, CD8^+^CD3e^+^ cytotoxic T cells, CD4^+^CD8^+^CD45RO^+^ memory T cells and CD8^+^CD45RO^+^TOX^+^ exhausted T cells. Notably, all T cell subsets in proximity to CLL cells contained substantial lipid accumulation (Fig. 4m,n). This pattern suggests that tumor-induced metabolic remodeling extends beyond malignant B cells to neighboring immune cells. Lipid accumulation in peritumoral T cells may indicate disrupted lipid homeostasis and potential impairment of effector functions and proliferative capacity.

Notably, in normal lymph nodes, CD68^+^ macrophages exhibited higher lipid levels and a lower redox ratio than the surrounding CD20^+^ B cells (Fig. 4q,r,s), indicating active oxidative metabolism, which was disrupted in lymphoma (Fig. 4t). These changes may aid the transition from M1 to M2 macrophages in the tumor microenvironment^35^. These results show that REDCAT enables high-resolution chemical profiling of individual cells in complex tissues, revealing region-specific and cell-type-specific metabolic reprogramming during lymphoma transformation.

### Super-resolved REDCAT illuminates subcellular lipid diversity and nuclear heterogeneity in lymphoma

**Fig. 5:**
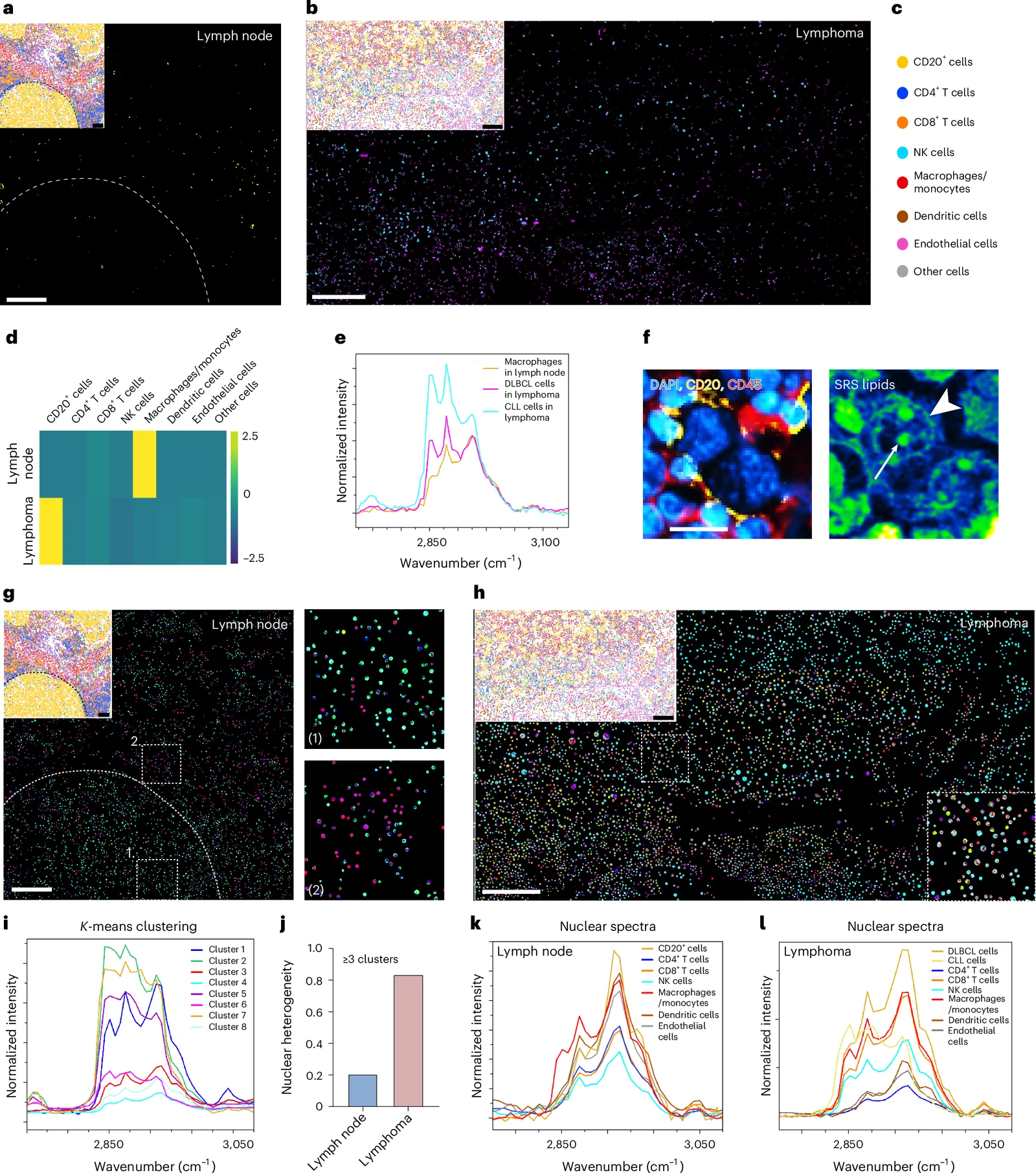
LD diversity and nuclear metabolic heterogeneity in normal lymph nodes and lymphoma. **a**, Lipid signal in the cytoplasm of cells from normal lymph node tissue showing lipid distribution; inset shows spatial cell-type mapping of the same ROI. Scale bars, 100 μm. **b**, Lipid signal in the cytoplasm of cells from lymphoma tissue showing lipid distribution; inset shows the spatial cell-type mapping of the same ROI. Scale bars, 100 μm. **c**, The different color dots indicate different cell types shown in **a** and **b**. **d**, Heat map showing the lipid level in each cell type of lymph nodes and lymphoma. The lipid level was normalized to the total lipids in the corresponding tissue ROI. **e**, Average SRS lipid spectra of LDs in macrophages in normal lymph node, CLL and DLBCL cells in lymphoma tissue. SRS spectra were processed by baseline subtraction and smoothing, followed by a two-step normalization strategy by preserving the intrinsic spectral shape and relative peak relationships within each spectrum as well as the inter-spectral intensity relationships. **f**, CODEX image (left) of an ROI showing several large B lymphoma cells and selected markers CD20 (yellow), DAPI (blue) and CD45 (red), alongside the SRS image (right) of lipid signal from the same ROI. The arrow indicates the nucleolus, and the arrowhead indicates the nuclear envelope. Scale bar, 10 μm. **g**, Spatial mapping of nuclear SRS spectra in normal lymph tissue; the inset shows spatial cell-type mapping of the same ROI. Right panels show magnified nuclear clusters inside (1) and outside (2) follicles. Scale bar, 100 μm. **h**, Spatial mapping of nuclear SRS spectra in lymphoma; the upper inset shows spatial cell-type mapping of the same ROI. The lower inset shows the magnified nuclear clusters. Scale bar, 100 μm. **i**, *k*-means clusters of nuclear SRS spectra shown in **g** and **h**. **j**, Quantification of the percentage of nuclei displaying ≥3 metabolic clusters in lymph nodes versus lymphoma. SRS spectra were processed by baseline subtraction and smoothing, followed by a two-step normalization strategy by preserving the intrinsic spectral shape and relative peak relationships within each spectrum as well as the inter-spectral intensity relationships. **k**,**l**, Cell-type-specific nuclear SRS spectra of lymph nodes (**k**) and lymphoma (**l**). SRS spectra were processed by baseline subtraction and smoothing, followed by a two-step normalization strategy by preserving the intrinsic spectral shape and relative peak relationships within each spectrum as well as the inter-spectral intensity relationships.

To investigate cytoplasmic lipid remodeling in lymphoma, we segmented the cytoplasm, identified pixels with high lipid content using SRS-HSI hyperspectral data and mapped the lipid signal to each cell type (Fig. 5a–c). In normal tissues, lipids were enriched in macrophages, with spectra peaking at 2,880 cm^−1^ (Fig. 5d,e). In lymphoma tissues, substantial lipids appeared in CD20^+^ cells (most CLL and DLBCL cells), with spectral peaks at 2,850 cm^−1^ and 2,880 cm^−1^ (Fig. 5d,e). These results align with the whole-cell spectral analysis shown in Fig. 4e.

Adaptive moment estimation (Adam) optimization-based pointillism deconvolution (A-PoD)-assisted SRS super-resolution processing^36^ illuminated lipid droplet (LD) structures by their density, lipid intensity and morphology. CLL cells had large LD clusters, whereas DLBCL cells had smaller LDs (Supplementary Fig. 1a), indicating different lipid storage regulations in tumor subtypes. Super-resolution analysis revealed elevated lipid signals in the nucleoli and (peri-)nuclear envelope of DLBCL cells (Fig. 5f and Supplementary Fig. 1b), suggesting that lipids may influence gene regulation or nuclear structural integrity. Both the nucleoli and nuclear envelope showed dominant lipid Raman peaks at 2,850 cm^−1^ and 2,880 cm^−1^, with distinct spectral profiles from each other and the cytoplasm, indicating a compartment-specific metabolic organization (Supplementary Fig. 1c).

Motivated by this observation, we profiled the nuclear metabolic signatures of normal lymph nodes and lymphoma tissues. The single-nuclear spectrum was extracted and subjected to *k*-means clustering (Fig. 5h–j). Compared to normal lymph nodes, lymphoma nuclei showed greater metabolic heterogeneity, with more clusters per nucleus (Fig. 5g–i). Over 80% of lymphoma nuclei contained more than three clusters, whereas only ~20% of nuclei in normal lymph nodes showed this level of complexity (Fig. 5j). Spatial mapping revealed that nuclei in follicles were enriched in cluster 4, while interfollicular regions were dominated by cluster 6, indicating that nuclear metabolic profiles can distinguish tissue features (Fig. 5g,i). This indicates that the nuclear spectra were associated with the cell type and subtype. We plotted the average nuclear spectrum for each cell type and observed distinct profiles between the cell types in both lymph nodes and lymphomas. Notably, each cell-type-specific spectrum was remodeled in lymphoma compared to lymph nodes, indicating profound alterations in nuclear metabolism with malignant transformation (Fig. 5k,l). These findings demonstrate that REDCAT enables tissue-wise exploration of lipid biology in subcellular compartments including LDs and nuclei.

### REDCAT unveils disorganized collagen architecture and altered composition in lymphoma

**Extended Data Fig. 8:**
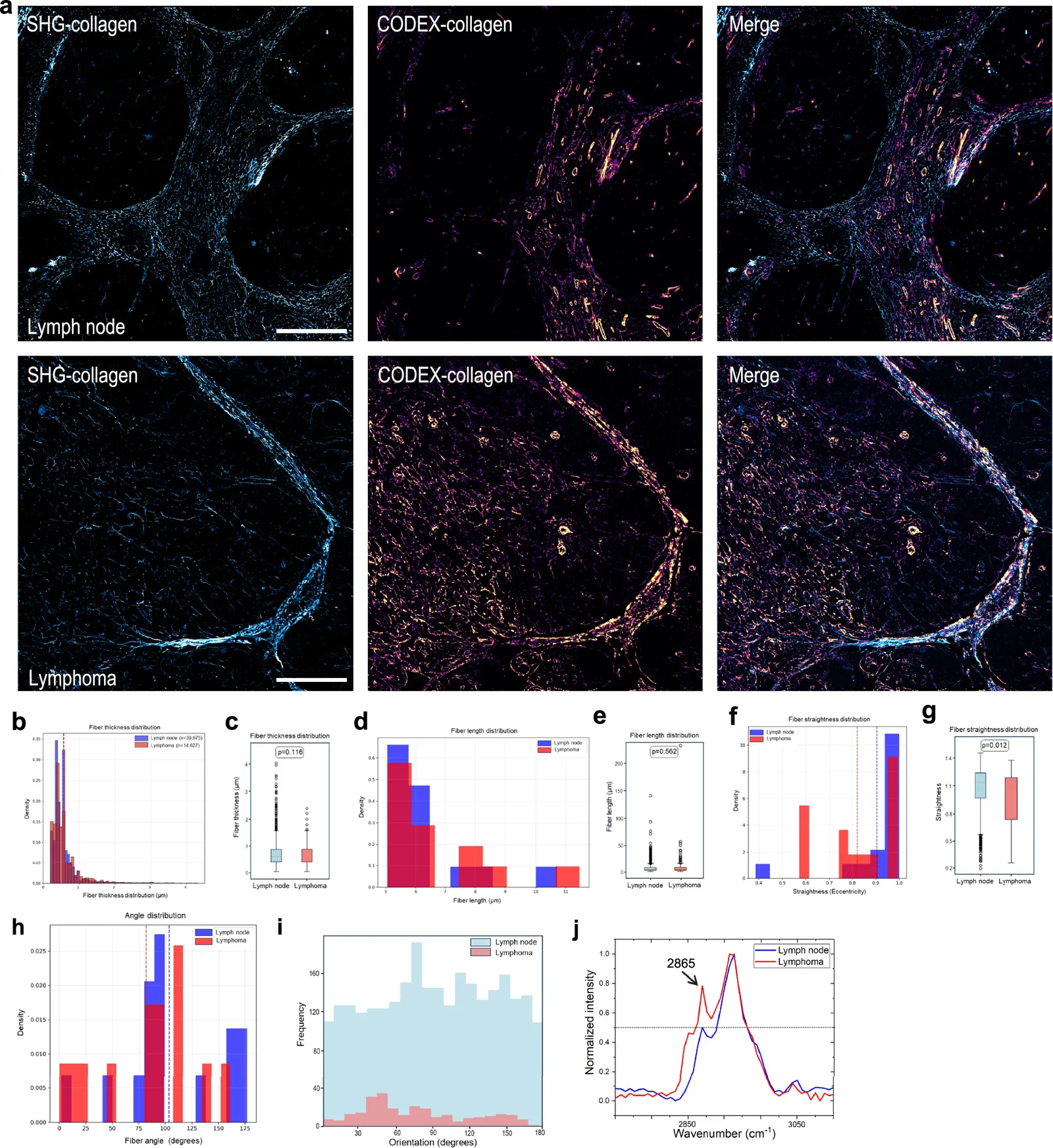
Collagen fiber architecture and composition differences between lymph node and lymphoma. **(a)** Representative SHG images of collagen fibers (type Ⅰ, Ⅱ, Ⅲ) in lymph node (top row) and lymphoma (bottom row) (left); Representative codex images of collagen fibers (type Ⅳ) in lymph node (top row) and lymphoma (bottom row) (middle); merged CODEX and SRS collagen channels (right). Scale bar, 200 μm. **(b, c)** Fiber thickness distribution histograms (**b**) and boxplots for mean fiber thickness (**c**) in lymph node and lymphoma. *p*-value was calculated using a two-tailed Student’s *t*-test. n = 3 ROIs. **(d, e)** Fiber length distribution histograms (**d**) and boxplots (**e**) comparing lymph node and lymphoma. *p*-value was calculated using a two-tailed Student’s *t*-test. n = 3 ROIs. **(f, g)** Fiber straightness distribution histograms (**f**) and boxplots (**g**) between lymph node and lymphoma. *p*-value was calculated using a two-tailed Student’s *t*-test. n = 3 ROIs. **(h)** Angle distribution density plots with mean angle indicated by dashed lines. **(i)** Histogram of collagen fiber orientation shows frequency distribution of angles in lymph node and lymphoma. **(j)** Average SRS spectra of collagen-rich regions from lymph node and lymphoma tissues. The dashed line was to mark the half-height bandwidths of collagen spectra.

In addition to subcellular metabolic profiling, we used REDCAT to analyze the extracellular matrix by integrating SHG images of collagen fibers (types I, II and III) and CODEX images of collagen fibers (type IV). As shown in Extended Data Fig. 8a, normal lymph nodes had densely distributed collagen fibers along the capsule and trabeculae, forming a well-organized network of collagen fibers. In contrast, the lymphoma samples showed reduced collagen density and a disorganized fiber network.

Collagen fibers in lymphoma were thinner (Extended Data Fig. 8b,c) and shorter (Extended Data Fig. 8d,e) than those in normal lymph nodes. Fiber straightness showed that lymphoma-associated fibers were more curved, whereas normal lymph nodes had straighter fibers (Extended Data Fig. 8f,g). Orientation analysis revealed broad angular dispersion in lymphoma, unlike the uniform alignment along the capsule and trabeculae in normal lymph nodes (Extended Data Fig. 8h,i).

SRS spectral profiling of collagen-rich regions uncovered clear biochemical differences between the two tissue types. Lymphoma samples displayed increased peak intensity at 2,865 cm^−1^, corresponding to asymmetric CH_2_ stretching features, along with an increased ~55.6 cm^−1^ of half-height bandwidth (Extended Data Fig. 8j), indicating changes in collagen composition and molecular packing. Together, these structural and compositional changes demonstrate that lymphoma is associated with profound remodeling of the extracellular matrix, characterized by collagen fiber loss, disorganization and altered molecular makeup, which may impact tissue integrity and tumor cell spread and extension into adjacent tissues.

### REDCAT mapping of subcellular metabolic activities in human liver

**Fig. 6:**
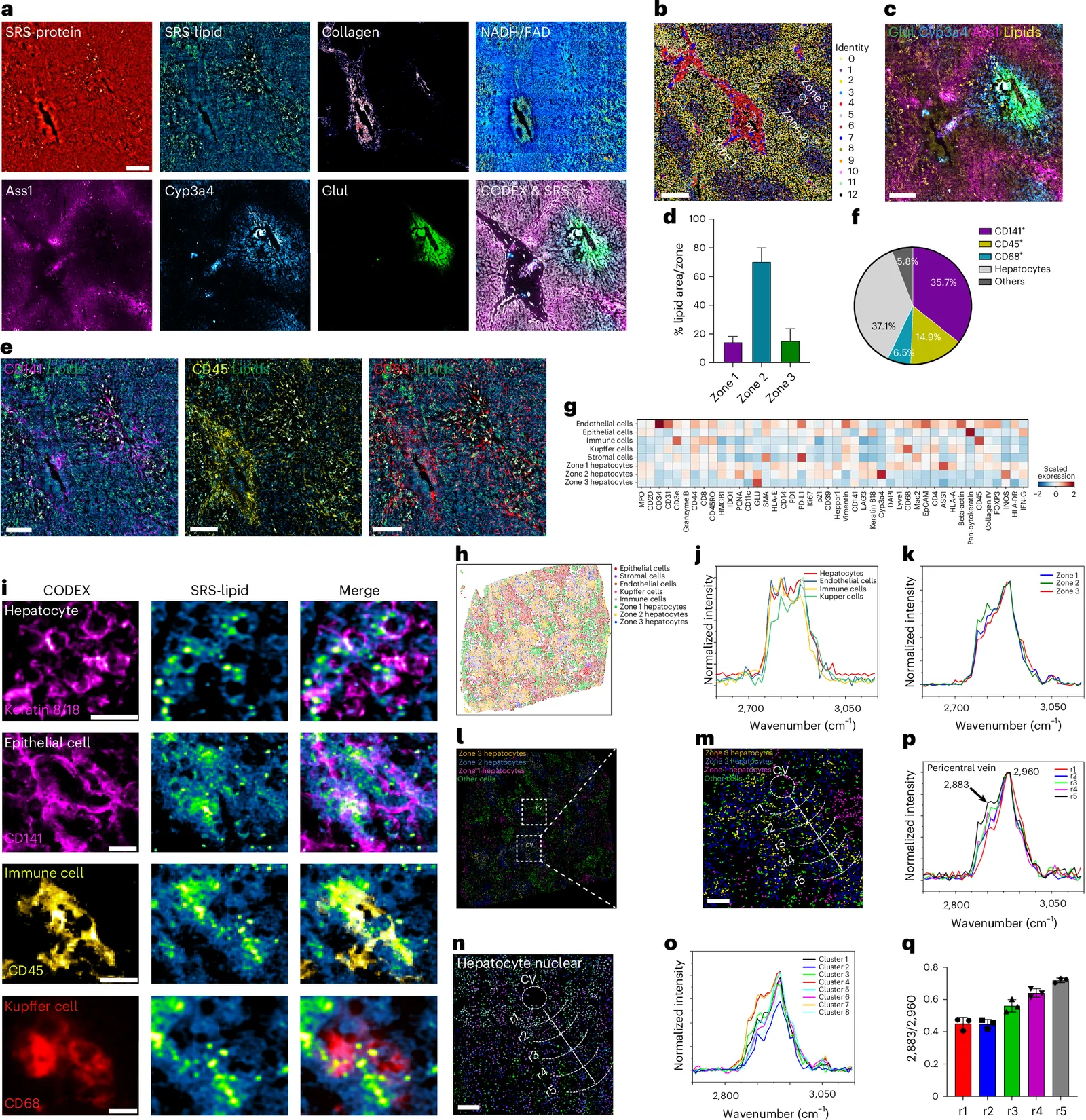
Spatial metabolic and phenotypic mapping of liver zonation using REDCAT. **a**, REDCAT multimodal imaging of liver tissue showing SRS protein, SRS lipid, SHG collagen, NADH/FAD redox ratio, immunofluorescence for Ass1, Cyp3a4 and Glul, and co-registered CODEX with SRS. Scale bar, 200 μm. **b**, CODEX clustering map overlaid on tissue architecture highlighting distinct liver zones. Scale bar, 200 μm. **c**, Spatial distribution of Glul, Cyp3a4, Ass1 and lipid channels mapped onto cell clusters. Scale bar, 200 μm. **d**, Quantification of lipid area fraction across liver zones. Data are shown as the mean ± s.e.m. to indicate the heterogeneity of lipid distribution across the different ROIs. *n* = 3 ROIs. **e**, Spatial colocalization of LDs with CD141, CD45 and CD68 immunostaining. Scale bars, 200 μm. **f**, Pie chart showing relative abundance of major cell populations (CD141^+^, CD45^+^, CD68^+^, hepatocytes, and others). **g**, Heat map of marker expression across annotated cell types in different liver zones. **h**, Spatial UMAP embedding of single-cell populations colored by cell type. **i**, Representative high-magnification images of hepatocytes, epithelial cells, immune cells and Kupffer cells showing cell-type-specific lipid distribution. Scale bars, 20 μm. **j**,**k**, Average SRS spectra of annotated cell types in liver zones (**j**) and from selected immune cell types (**k**). SRS spectra were processed by baseline subtraction and smoothing, followed by a two-step normalization strategy by preserving the intrinsic spectral shape and relative peak relationships within each spectrum as well as the inter-spectral intensity relationships. **l**,**m**, Overview image of the distribution of hepatocytes from different zones (**l**) highlighting the pericentral area shown in **m**. Curved lines delineate concentric regions (r1–r5) extending outward from the central vein at ~100-µm intervals, marking nuclei within each zone for spatial classification of hepatocytes. Scale bar, 100 μm. **n**, Nuclear spatial maps of selected cell populations shown in **m**. Scale bar, 100 μm. **o**, *k*-means clusters of SRS spectra from nuclei in liver tissue. SRS spectra were processed by baseline subtraction and smoothing, followed by a two-step normalization strategy by preserving the intrinsic spectral shape and relative peak relationships within each spectrum, as well as the inter-spectral intensity relationships. **p**, SRS spectra of indicated regions shown in **n**. SRS spectra were processed by baseline subtraction and smoothing, followed by normalization to peak intensity at 2,960 cm^−1^. **q**, Quantification of 2,883 cm^−1^/2,960 cm^−1^ ratio based on SRS spectra across ROIs near the central vein in **n**. Data are shown as the mean ± s.e.m. to indicate the trend of intensity ratio changes near the central vein region. *n* = 3 different central vein regions. CV, central vein; PV, portal vein. Source data

**Extended Data Fig. 9:**
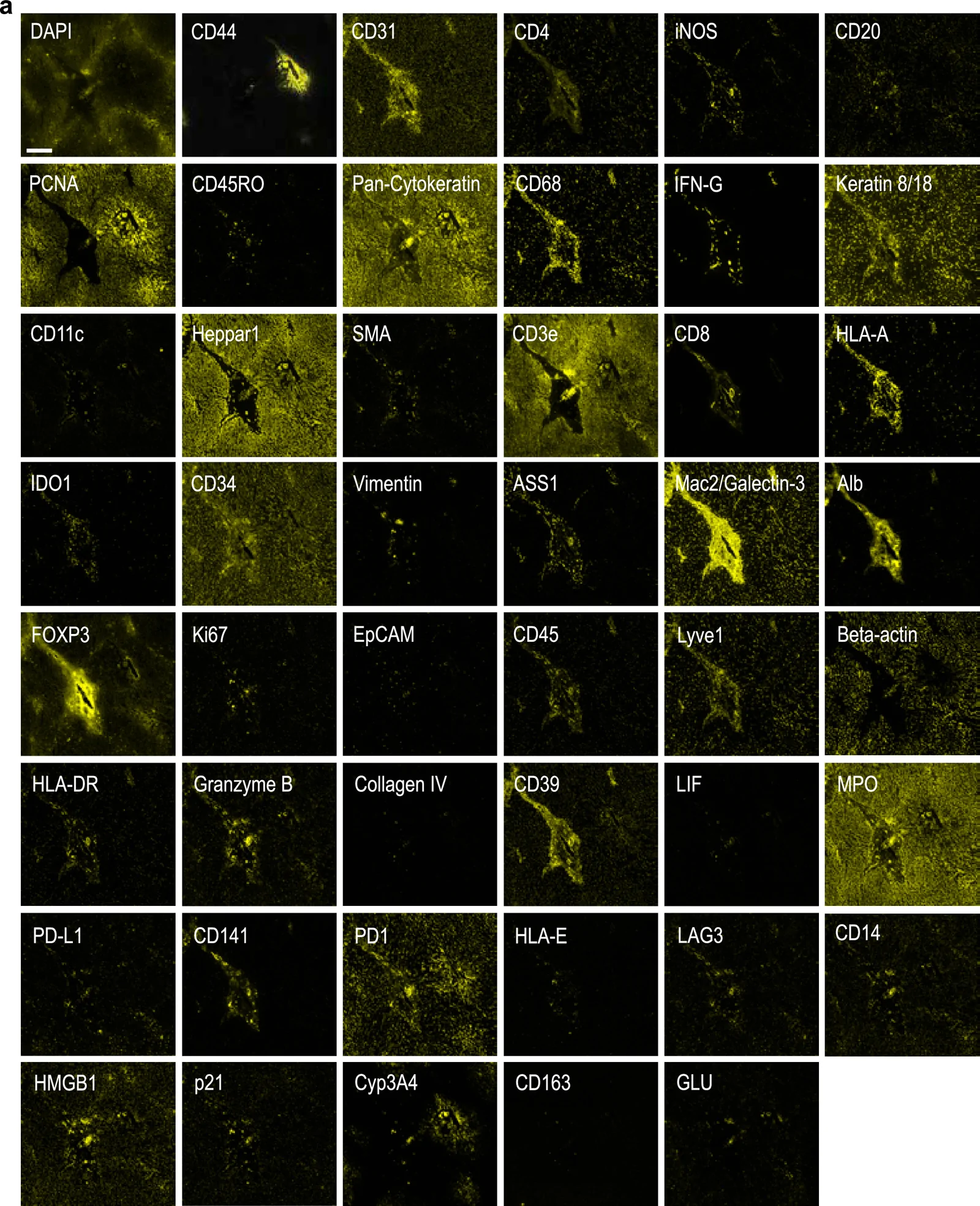
CODEX antibody panel validation for liver tissue. **(a)** Representative single-channel images showing staining patterns for structural, immune, stromal, metabolic, and functional markers in liver tissue. Markers include nuclear and structural proteins (DAPI, PCNA, Beta-actin, Collagen IV, Vimentin), immune lineage markers (for example, CD3e, CD4, CD8, CD11c, CD14, CD20, CD31, CD44, CD45, CD45RO, CD68, CD141, CD163), hepatic and metabolic enzymes (for example, HepPar1, ASS1, Cyp3A4, GLUL, Alb), stromal/ECM markers (SMA, Pan-Cytokeratin, Keratin 8/18, Lyve1, Mac2/Galectin-3), functional and activation markers (Ki67, FOXP3, IFN-γ, Granzyme B, LAG3, PD-1, PD-L1, p21, MPO, HMGB1, IDO1, LIF, iNOS), and MHC molecules (HLA-A, HLA-DR, HLA-E). All images were acquired under identical conditions to ensure direct comparison of staining patterns and intensities. Scale bar, 200 μm.

**Extended Data Fig. 10:**
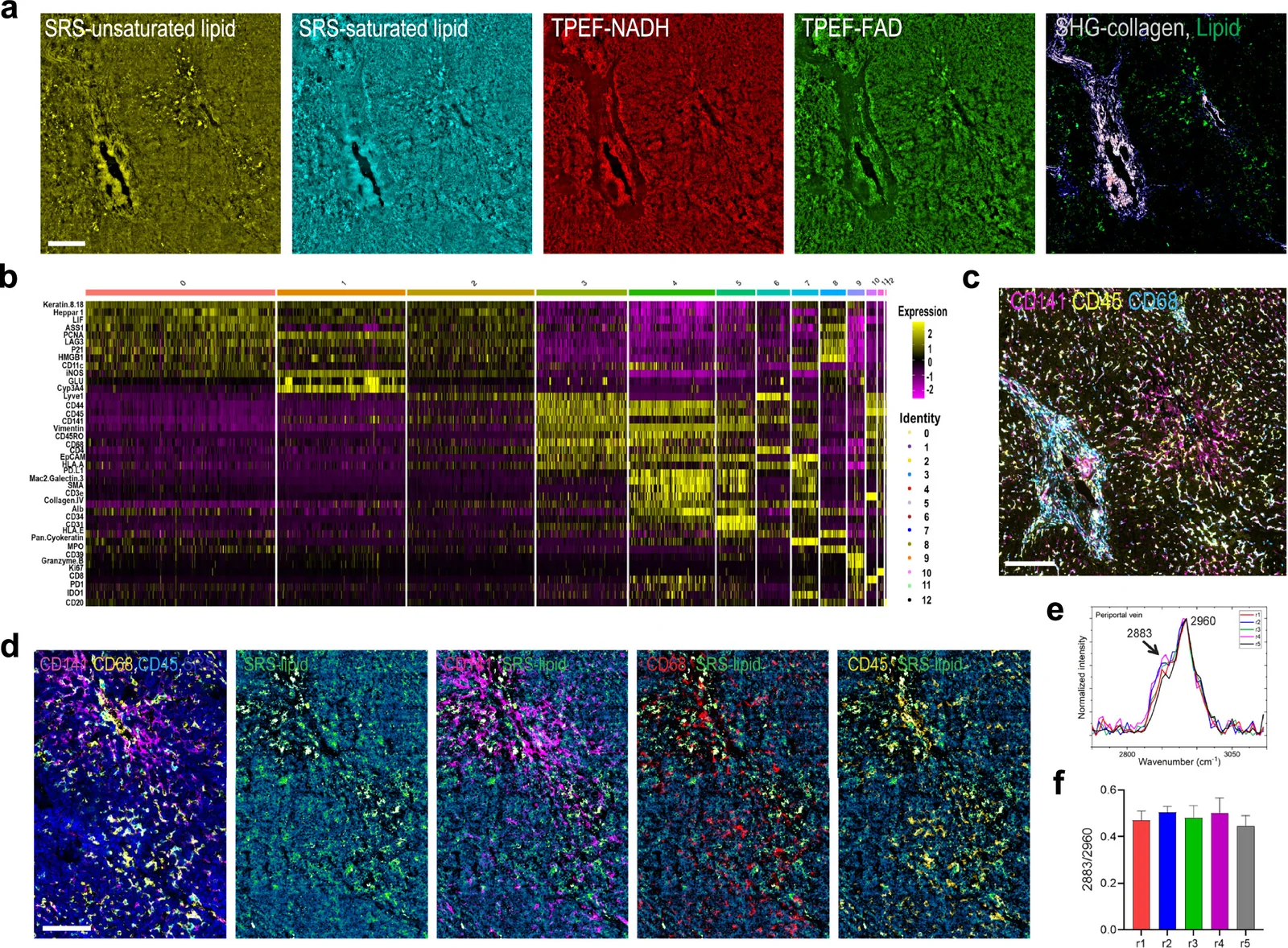
Integrated metabolic and proteomic profiling of liver tissue microenvironments. **(a)** Multimodal imaging channels showing SRS-unsaturated lipid, SRS-saturated lipid, TPEF-NADH autofluorescence, TPEF FAD autofluorescence, and SHG-collagen with overlaid SRS-lipid channel. Scale bar, 200 μm. **(b)** Heatmap of scaled protein marker expression across 12 cell clusters identified by CODEX, with cluster identities indicated above. **(c)** Spatial CODEX map showing localization of CD141 (magenta), CD46 (yellow), and CD68 (cyan) expressing cells in the same ROI. Scale bar, 200 μm. **(d)** CODEX images of another selected region showing overlays between CD141, CD68, and CD45 with SRS-lipid channel (left), and individual SRS lipid channel, and individual CODEX channels for CD141, CD68, and CD45. Scale bar, 200 μm. **(e)** Mean SRS spectra from five representative regions of interest (r1–r5) near PV (indicated in Fig. 7l, PV). Concentric regions (r1–r5) extending outward from the PV at 100 µm intervals, marking nuclei within each zone for spatial classification of hepatocytes. SRS spectra were processed by baseline subtraction and smoothing, followed by normalization to peak intensity at 2960 cm^−1^. **(f)** Quantification of the 2883 cm^−1^/2960 cm^−1^ peak ratio across r1–r5 near portal vein (PV). Data was shown as mean ± SEM to indicate the trend of intensity ratio changes near PV region. n = 3 different PV regions. Source data

To demonstrate REDCAT’s ability to resolve metabolic heterogeneity in similar cell types and its compatibility with fresh-frozen tissues, we applied REDCAT to a fresh-frozen normal human liver tissue section (Fig. 6 and Extended Data Figs. 9 and 10). The liver is organized into hexagonal lobules. Blood flows into each lobule via the hepatic artery and portal vein in the periportal region, draining toward the central vein in the pericentral region. The liver shows functional zonation based on metabolic functions, with hepatocytes in zones 1 (periportal), 2 (midzone) and 3 (pericentral) differing metabolically^37,38^. scRNA-seq has shown that these gradients lead to the expression of distinct metabolic genes, and electron microscopy has revealed notable differences in mitochondrial morphology between periportal and pericentral hepatocytes^37^. However, the functional implications of these structural and molecular differences are not yet fully understood.

To assess if metabolic variations correlated with redox ratio and lipid profiles’ spatial positioning, TPEF–SRS imaging and 47-plexed CODEX imaging were applied to the liver ROI (1.6 mm × 1.6 mm), including both central and portal veins (Fig. 6a and Extended Data Figs. 9a and 10a). Unsupervised clustering of CODEX data identified 13 clusters (Extended Data Fig. 10b), and these clusters were spatially organized to the physiological architecture (Fig. 6b), evidenced by the zonation markers arginase synthesis 1 (ASS1)/zone 1, cytochrome P450 3A4 (Cyp3A4)/zone 2 and glutamine synthetase (GS or Glul)/zone 3 (Fig. 6a). TPEF–SRS imaging revealed uneven LDs and NADH/FAD ratio distribution among the three zones (Fig. 6a,c and Extended Data Fig. 10a), consistent with literature on hepatocytes’ exposure to varying oxidative stress and redox states based on lobule location. LD levels and redox ratio were notably lower in zone 1 compared to areas near the central vein (Fig. 6a,c and Extended Data Fig. 10a). Quantifying LD area showed they were mainly in zone 2 (Fig. 6d). By registering LD distribution with cell-type-specific markers, we observed that LDs were present not only in hepatocytes expressing ASS1, Cyp3A4 and Glul (37.1%), but also in CD141^+^ (35.7%), CD45^+^ (14.9%) and CD68^+^ (6.5%) cells (Fig. 6c,e,f). Single-cell annotation and spatial mapping (Fig. 6g,h) identified these cells as epithelial, immune and Kupffer cells, respectively. These lipid-laden cells were mainly situated near the liver sinusoids, forming part of the immune cell landscape of the liver (Extended Data Fig. 10c). This cell-type-specific LD localization was confirmed in another ROI (Extended Data Fig. 10d).

To investigate cell-type-specific LD composition, we acquired SRS-HSI data and conducted chemical profiling on individual LDs. The results revealed that LDs from different cell types exhibited distinct spectral signatures (Fig. 6i,j), suggesting that LD diversity may be critical for defining cell identity and supporting immune function. However, the signaling pathways regulating lipid metabolism within individual cell types, as well as their impact on liver function, remain to be elucidated. Notably, we also observed differences in the SRS spectral profiles of hepatocytes across liver zonation (Fig. 6k), indicating metabolic heterogeneity associated with spatial location and metabolic state even in the same cell type.

Previous spatial single-nuclear metabolomics studies^12^ have shown that the pericentral region is particularly enriched in glucose-related metabolites, with concentrations gradually decreasing along the proximal-to-distal axis from the central vein. To evaluate whether REDCAT can similarly resolve zonation-dependent nuclear metabolic heterogeneity, we examined the metabolic profiles of individual nuclei within a ROI located near the central vein (Fig. 6l,m). SRS-HSI followed by unsupervised clustering identified eight distinct SRS spectral clusters (Fig. 6n,o). Gradient orientation analysis revealed distance-dependent changes in SRS spectra (Fig. 6p), and quantitative analysis further showed that the 2,883 cm^−1^/2,960 cm^−1^ ratio increased from the central vein toward the outer region (Fig. 6q). However, this phenomenon was not detected near the periportal region (Extended Data Fig. 10e,f), suggesting a region-specific metabolic organization.

Collectively, these findings demonstrate that REDCAT can comprehensively characterize cell-type-specific LD diversity and spatial heterogeneity in redox states, lipid distribution and nuclear metabolic profiles within fresh-frozen liver tissues.

## Discussion

We developed REDCAT, a multimodal optical platform integrating SRS chemical imaging and CODEX immunofluorescence imaging, to examine proteomics and metabolic profiles at the single-cell level in tissues, including FFPE and fresh-frozen samples. In FFPE lymph nodes and lymphoma tissues, REDCAT monitors metabolic reprogramming during immune cell activation and identifies subcellular metabolic features in tumor development. Our data indicate lipid species are preserved in FFPE tissues as cellular structures retain small molecules during fixation. Thus, REDCAT’s capability to explore lipid biology in FFPE tissues holds great potential in clinical histopathology research.

In normal lymph node tissue, lipid levels negatively correlate with NADH/FAD ratios across cell types. This suggests lipid amounts and redox states could indicate immune cell metabolic homeostasis in human lymph nodes. However, this correlation is disrupted in lymphoma tissues. In CLL and DLBCL cells, global lipid levels, redox ratio and nuclear heterogeneity are upregulated, indicating substantial metabolic remodeling. Tumor cells may engage metabolic gene expression and pathways in catabolism to upregulate NADH for hypoxic stress adaptation while synthesizing lipids for energy and cell building. Lipids may also result from reduced lipogenesis or environmental scavenging. In CLL, the substantial LD accumulation is consistent with CD36 protein expression reported previously^39^. Additionally, lysosome abundance is reported to be higher in CLL than normal B cells^39^, potentially participating in the lipid processing through autophagy. Alternatively, toxic lipids, such as oxidative lipids from membranes or external sources, can be sequestered in LDs, protecting cells from lipotoxicity^40,41^. However, the metabolic routes of these lipids need further validation. Combining isotope-probed SRS imaging^25,42^ with genome-wide spatial omics profiling such as Patho-DbiT^6^ to explore specific lipid metabolism genes in different cell types and transcriptional states can help decipher the source of the lipids and the mechanisms regulating LD generation in normal physiology or tumorigenesis. Multiple lipid metabolic pathways and collagen remodeling-associated metabolic pathways were markedly disturbed within the same lymphoma sample in our DBiTplus study^43^.

We demonstrated unique LD morphology and metabolic profiles linked with CLL and DLBCL cell subpopulations. CLL-to-DLBCL transformation involves lipid distribution remodeling, including nuclear and membrane lipids. DLBCL often associates with chronic inflammation from inflammatory environments or Epstein–Barr virus infection^44^. This DLBCL sample was positive for Epstein–Barr virus. Inflammatory sites allow B cells to acquire genetic changes promoting lipid metabolic reprogramming, priming tumorigenesis. Our exploratory findings suggest targeting abnormal metabolic processes may prevent tumor progression. CLL and surrounding cells, like endothelial, T, NK and dendritic cells, accumulate more lipids than distant cells, indicating dynamic metabolic remodeling during tumor transformation. Cancer cells may engage with surrounding cells in metabolic modulation, impairing antitumor immunity or promoting a tumor environment. Tumor cells may release exosomes with metabolites absorbed by adjacent cells^45^. These exosomes can reprogram endothelial cells to produce cytokines supporting CLL cell survival and proliferation. Lipid accumulation was detected within DLBCL cell nucleoli. Previous studies show lipids in mammalian cell nucleoli^46^. Nucleolar protein RRP-8 suppression is linked to abnormal lipid accumulation in nuclei by inducing nucleolar stress^47^. Nucleolar lipids may be involved in signaling, regulating gene expression and maintaining nucleolar structural integrity during B cell tumor transformation, but their functions require further investigation. Other tumor cell types may exhibit lipodystrophy. In normal macrophages, lipids in CD68-positive vesicles likely originate from endocytosed cell debris, indicating active immune surveillance. However, in lymphoma-infiltrated macrophages, lipid levels were reduced, suggesting dysfunction in debris processing and compromised lysosomal metabolic flux. Investigating lipid metabolism across tumor niche cell types is essential for understanding tumor pathology.

Similar cell types dependent on LD diversity in size and composition have been found in fresh-frozen healthy liver tissues. We found LDs in both parenchymal and non-parenchymal hepatic cells, including immune cells such as Kupffer cells and resident macrophages involved in immune surveillance. The liver, a major immunological organ, monitors the blood from the gastrointestinal tract via the portal vein. Its immune system balances tolerance to harmless antigens with rapid responses to pathogens and injuries. LDs in immune cells may play a role in this process. However, the regulation of specific lipid profiles in each cell type and the contribution of lipid diversity to immune function require further investigation. We also found that nuclear chemical properties showed distance-dependent changes from proximal to distal near the central vein region, possibly related to the genome opening status for gene expression. Exploring nuclear components or structures representing these spectral changes may clarify the mechanisms underlying spatial gene expression and compartmentalized liver function.

Some challenges remain in metabolite annotation and coverage using REDCAT. These limitations can be addressed by incorporating hyperspectral acquisition in the fingerprint region, expanding reference libraries for diverse metabolites, and applying generative AI-assisted data analysis. One of REDCAT’s strengths lies in its ability to identify tissue histological patterns distinguished by high-resolution metabolic features, serving as essential clues for subsequent metabolite identification using the annotation tool. In addition, REDCAT can be integrated with other spatial technologies and is not restricted to any specific MSI platform, broadening its applicability across spatial metabolomics profiling.

In summary, REDCAT offers a high-spatial-resolution, single-cell-type-specific metabolic profiling and analysis pipeline that is label-free and requires minimal sample preparation. It adapts to diverse biological samples, from cell cultures to complex tissues, and can uncover subtle metabolic alterations that are undetectable or complementary to conventional assays. With advances in SRS resolution, spectral coverage and metabolite annotation, REDCAT will enable more detailed single-cell or subcellular spatial metabolomic mapping, providing deeper functional and mechanistic insights into biological processes.

## Methods

### Tissue specimens

De-identified archived human lymphoid tissue samples were obtained from Yale Pathology Tissue Services (YPTS). The tissue retrieval and distribution for research were conducted with the approval of the Yale University Institutional Review Board (approved IRB, 1401013259) and oversight by the Tissue Resource Oversight Committee. Written informed consent for participation in any case where identification was collected alongside the specimen, was obtained from participants or their guardians, in accordance with the principles of the Declaration of Helsinki. Each sample was handled in strict compliance with Health Insurance Portability and Accountability Act (HIPAA) regulations, university research policies, pathology department diagnostic requirements and hospital by-laws.

### Human tissue section preparation

After all slides were reviewed by a board-certified pathologist and the optimal block was selected, paraffin blocks were sectioned at a thickness of 7–10μm and mounted on the center of poly-L-lysine-coated 1 × 3-inch glass slides. Serial tissue sections were collected simultaneously for TPEF–SRS, CODEX and H&E staining. The sectioning was carried out by YPTS and stored at −80 °C until use.

### FFPE tissue information and deparaffinization

The normal lymph node tissue section was acquired from an individual with a body mass index value of 32, and the tumor tissue section was collected from an individual with a body mass index value of 37. The FFPE and fresh-frozen tissue sections were processed at Yale University and shipped to UCSD for multimodal SRS imaging. The FFPE tissue slides were bathed with fresh xylene for 5 min and then were transferred to fresh absolute ethanol for 5 min. Following sequential incubation in 95%, 75%, 50% and 25% ethanol for 3 min each, the slides were immersed in distilled water for 3 min for full hydration.

### Liver tissue de-crosslinking

The fresh-frozen healthy liver tissue section on the PLL slide was processed through the AKOYA-PhenoCycler-Fusion fresh-frozen Tissue Pre-Antibody station. The slide was placed on Drierite beads for 5 min and placed in acetone for 10 min, and the slide was held at room temperature for 2 min. Then the slide was immersed for 2 min in hydration buffer 1 and the same step was repeated in hydration buffer 2. Finally, the tissue on the slide was fixed by immersing in 1.6% paraformaldehyde for 10 min. This fixated tissue slide was shipped to UCSD in a centrifuge tube filled with hydration buffer at 4 °C.

### SRS imaging and high-resolution imaging processing

The SRS images were collected from an upright laser-scanning microscope (DIY multiphoton, Olympus), which was equipped with a ×25 water objective (XLPLN, WMP2, 1.05 NA, Olympus). The synchronized pulsed pump beam (tunable 720‒990-nm wavelength, 5–6-ps pulse width and 80-MHz repetition rate) and stokes beam (wavelength at 1,031 nm, 6-ps pulse width and 80-MHz repetition rate) from a picoEmerald system (Applied Physics & Electronics) were coupled and introduced into the microscope. Upon interacting with the samples, the transmitted signal from the pump and Stokes beams was collected by a 1.4-NA oil condenser. A short-pass filter (950 nm, Thorlabs) was used to completely block the Stokes beam and transmit the pump beam only onto a silicon photodiode array for detecting the stimulated Raman loss signal. The current output from the photodiode array was terminated, filtered and demodulated by a lock-in amplifier at 20 MHz. The demodulated signal was fed into the FV3000 software module FV-OSR (Olympus) to form images using laser scanning. SRS protein, lipid, unsaturated lipid and saturated lipid signals were obtained at vibrational modes 2,930 cm^−1^, 2,850 cm^−1^, 2,880 cm^−1^ and 3,015 cm^−1^, respectively, in a single tile of 512 × 512 pixels, at dwell time of 40 μs, and 100 tiles were stitched to generate the large tissue mapping. To quantify the unsaturated lipid ratio, the intensity of C = C vibrational modes (peak intensity at 3,015 cm^−1^) was divided by the intensity of saturated vibrational modes (peak intensity at 2,880 cm^−1^), resulting in ratiometric images. Minor intensity adjustment (brightness and/or contrast) was performed in ImageJ. The figures were assembled in Adobe Illustrator 2023. To reach a high resolution of the subcellular organelles, the measured images can be processed by A-PoD. Based on a function to describe the blurring pattern of point emitters, the so-called point spread function, the super-resolution images can be reconstructed.

### SRS hyperspectral imaging and clustering analysis

SRS hyperspectral images were collected from the same setup described above. The hyperspectral imaging stacks were taken with 51 spectral steps at 40-μs pixel dwell time, covering the whole C–H stretching region from 2,800 cm^−1^ to 3,150 cm^−1^. The intensity profiles of the hyperspectral stacks from the ROIs were plotted in ImageJ and further processed by baseline correction, smoothing and normalization in OriginPro 2017. SRS hyperspectral imaging of 50 mM rhodamine 6G dye solution was performed in each imaging session for laser power calibration. The spectrum of rhodamine 6G was divided by all the sample spectra to obtain intensity-normalized SRS spectra for quantification purposes. An unsupervised *k*-means clustering method was applied to generate metabolic mapping at each pixel. SRS spectra were normalized by a two-step strategy. First, each individual spectrum was normalized to its highest-intensity peak by dividing the entire spectrum by the corresponding maximum intensity value, thereby preserving the intrinsic spectral shape and relative peak relationships within each spectrum. Second, to maintain consistent intensity relationships across distinct spectra within the same tissue, the maximum intensity value of each normalized spectrum was further scaled relative to the largest maximum intensity observed across all spectra from that tissue. This second step preserves inter-spectral intensity relationships within the tissue while minimizing variability due to acquisition-related fluctuations. The normalized SRS spectra were used to display in the pixel-wise clustering and regional and cell-type-specific spectra figures.

### Optical redox imaging

Two-photon fluorescence microscopy was used to collect the NADH and FAD signals from the tissue. The NADH signal was excited by 780 nm, and the emission wavelength was collected at 460 nm. The FAD signal was excited by 860 nm, and the emission wavelength was collected at 515 nm. The ratiometric images were generated by NADH/FAD. The ratio was further quantified in ImageJ and plotted in GraphPad 10.

### CODEX spatial phenotyping using PhenoCycler-Fusion

After receiving the slides following the SRS workflow, a modified version of the CODEX PhenoCycler-Fusion protocol (https://www.protocols.io/view/akoya-biosciences-phenocycler-fusion-formerly-code-x54v9p4j1g3e/v1/) was adopted. Since the tissue had already been deparaffinized and rehydrated during the SRS workflow, the CODEX process began with a gentle antigen-retrieval step using 1× AR9 buffer for 5–10 min. The tissue was then allowed to cool to room temperature and was rinsed twice with nuclease-free water and hydration buffer, followed by staining buffer as the antibody cocktail was prepared. The tissue slide was incubated with the antibody cocktail at room temperature for 3 h in a humidity chamber. After incubation, the tissue underwent a series of steps including post-fixation, ice-cold methanol incubation and a final fixation step. Attached to the flow cell, the tissue section was incubated in 1× PhenoCycler buffer with additive for at least 10 min to improve adhesion. The CODEX cycles were then set up, the reporter plate was prepared and loaded, and the imaging process began. A final .qptiff file was generated at the end, which could be viewed using QuPath v0.5.168. For further details on the PhenoCycler antibody panels, experimental cycle design and reporter plate volumes, see https://github.com/yal026/REDCAT/tree/main/CODEX/REDCAT/.

### Flow cell removal and H&E staining

Following the CODEX imaging workflow, the flow cell was removed, and histological H&E staining performed on the same tissue section. To remove the flow cell, the tissue slide with the flow cell was immersed in xylene or HistoClear for a minimum of 20 min to weaken the adhesive. A razor was then used to carefully detach the flow cell from the tissue slide. The tissue slide was then rinsed thoroughly with deionized water and then with 1× PhenoCycler Buffer without additive three times for 10 min each. Histological H&E staining on the FFPE sections was conducted by YPTS.

### Preprocessing of CODEX data

Cell segmentation was performed using a StarDist^47^-based model in QuPath^48^. The DAPI channel served as the nuclear marker for cell segmentation. The mean intensity of each marker for each segmented cell was exported together with the centroids of each cell as a CSV file. Downstream analysis was performed using Seurat 4.3.0 package. The dataset was normalized and scaled using NormalizeData and ScaleData functions. Linear dimensionality reduction was then performed with the ‘RunPCA’ function. The FindNeighbors function embedded spots into a *k*-nearest-neighbor graph structure based on Euclidean distance in PCA space, and the FindClusters function was used to cluster the spots. The RunUMAP function was used to visually show spatial heterogeneities through the UMAP algorithm. The clusters were plotted spatially using the ImageDimPlot function. The FindAllMarkers function was used to find the differentially expressed proteins in each cluster, and the heat map was plotted using the DoHeatMap function.

### Quality control and preprocessing for single-cell analysis

Cell and nuclei boundaries were generated using Mesmer^49^ with pretrained weights. The DAPI channel was used to identify nuclei, while the CD45 channel marked the cell membranes. The default resolution of 0.5 μm per spot was used for cell mask prediction. Only cells with sizes falling within the (0.05, 0.95) quantile range and exhibiting DAPI intensities above the 0.1 quantile threshold were included in the analysis. CODEX feature extraction involved summing the signal for each feature across individual cells. For each feature, the 0.05 and 0.95 quantiles were computed, and the values were normalized to fit within a (0, 1) scale, where 0 corresponded to the 0.05 quantile and 1 to the 0.95 quantile. Values outside this range were capped at 0 or 1, as needed.

### Cell-type annotation

Cell-type annotation was performed using the Astir package^50^. Cleaned and normalized protein expression matrices were used as input, ensuring high-quality data for downstream analysis. For Figs. 3–5, marker genes used in annotation included B cells (CD45, CD20, CD21), CD4^+^ T cells (CD45, CD3e, CD4), CD8^+^ T cells (CD45, CD3e, CD8), NK cells (CD45, CD107a), macrophages (CD45, CD68, CD163), monocytes (CD45, CD14), dendritic cells (CD45, CD141) and endothelial cells (CD31). For Fig. 6, marker genes used in annotation included zone 1 hepatocytes (ASS1, HLA-A), zone 2 hepatocytes (Cyp3a4), zone 3 hepatocytes (GLU), epithelial cells (EpCAM, Pan-Cytokeratin), stromal cells (PD-L1), endothelial cells (Lyve1, CD31, CD34), Kupffer cells (CD68) and immune cells (CD45). The annotation process was initialized with the fit_state() function, using default parameters to estimate an initial probabilistic state for each cell. The fit() function was then applied to train the model, which estimates the posterior probability of each cell belonging to predefined cell types based on marker expression patterns. Cells were annotated with the type showing the highest posterior probability. After cell annotation, SRS single cells were mapped onto their matched CODEX cells, and the metabolic profiles were extracted for analysis.

### MaxFuse integration of CODEX and scRNA-seq data

For Fig. 2j,n, we obtained the human lymph node scRNA-seq dataset from the Cell2location repository^51^ (available for download at https://cell2location.cog.sanger.ac.uk/browser.html). Before integration, all scRNA-seq data underwent standard preprocessing in ScanPy, including library-size normalization, log1p transformation and selection of highly variable genes, retaining the 5,000 most variable genes. Integration of the scRNA-seq and CODEX modalities was performed with the MaxFuse algorithm^16^. Cross-modal feature links were defined by matching protein and gene names; among these, features with standard deviation > 0.01 were kept enhancing integration performance. For pivot matching, the number of principal components used to construct the nearest-neighbor graph was chosen using the elbow criterion on the singular-value decomposition (SVD). To accommodate the weak linkage between modalities, a smoothing weight of 0.3 was applied as recommended by MaxFuse^16^. After integration, scRNA-seq cells were mapped onto their matched CODEX cells and visualized in spatial plots.

### SRS single-cell analysis

Fixed tissue sections were imaged by multimodal SRS microscopy and CODEX sequentially. Because fixation and gentle handling minimize mechanical drift, we assumed no appreciable nonrigid deformation during imaging. Any residual field-of-view shifts, rotations and uniform scaling were treated as global rigid/affine effects and corrected computationally. To co-register the modalities, we manually selected a set of visually reliable control points in the SRS and CODEX images. Given the two coordinate arrays, we estimated an optimal affine transformation by least squares using skimage.transform.estimate_transform(), which minimizes the sum of squared distances between matched control points. The SRS image was mapped into the CODEX coordinate system by applying the inverse transform with skimage.transform.warp(), yielding pixel-level alignment suitable for downstream cell-based analysis. Mapped cell centroids were then generated accordingly so that per-cell measurements could be compared one-to-one across SRS and CODEX. At the single-cell level, we computed ratio metrics reflecting metabolic and biochemical state, including NADH/FAD (redox balance), lipid/protein (overall lipid enrichment), and unsaturated-to-saturated lipid (degree of lipid unsaturation). For each metric, we summarized the upper tail by the 0.8 quantile to capture high-value distributions while remaining robust to occasional extremes. Scatterplots were generated to visualize the spatial distribution of the computed ratios across the tissue. In practice, affine alignment is generally sufficient for fixed sections acquired in close succession, providing a transparent, low-variance correction. Subtle local warping at tissue edges or folds may benefit from higher-order models, which could be worth exploring in cases with pronounced distortion.

### Reporting summary

Further information on research design is available in the Nature Portfolio Reporting Summary linked to this article.

## Online content

Any methods, additional references, Nature Portfolio reporting summaries, source data, extended data, supplementary information, acknowledgements, peer review information; details of author contributions and competing interests; and statements of data and code availability are available at https://doi.org/10.1038/s41592-026-03180-0.

## Supplementary information

Supplementary Information Supplementary Fig. 1.

Reporting Summary

## Source data

Source Data Fig. 2 Source data for Fig. 2 (FAD).

Source Data Fig. 2 Source data for Fig. 2 (NADH).

Source Data Fig. 2 Source data for Fig. 2 (lipid).

Source Data Fig. 2 Source data for Fig. 2 (protein).

Source Data Fig. 3 Source data for Fig. 3 (FAD).

Source Data Fig. 3 Source data for Fig. 3 (NADH).

Source Data Fig. 3 Source data for Fig. 3 (lipid).

Source Data Fig. 3 Source data for Fig. 3 (protein).

Source Data Fig. 6 Source data for Fig. 6d.

Source Data Fig. 6 Source data for Fig. 6f.

Source Data Fig. 6 Source data for Fig. 6q.

Source Data Extended Data Fig./Table 10 Statistical source data.

## Data Availability

The data supporting the findings of this study are available on Zenodo via https://doi.org/10.5281/zenodo.21938834 (ref. ^52^). SRS hyperspectral data are available from the corresponding author upon reasonable request. Source data are provided with this paper.

## Code availability

The code used for data analysis in this study is available on GitHub via https://github.com/yal026/REDCAT/.
