# Supplementary material for: All-optical multimodal mapping of single-cell-type-specific metabolic activities via REDCAT
Source: Nat Methods. 2026 Aug 27;23(9):1840–53. doi: 10.1038/s41592-026-03180-0 (PMC13541623; doi:10.1038/s41592-026-03180-0)
Supplement: Supplementary file 1 [file 41592_2026_3180_MOESM1_ESM.pdf]

nature methods

Supplementary information https://doi.org/10.1038/s41592-026-03180-0

All-optical multimodal mapping of single-cell-type-specific metabolic activities via REDCAT

In the format provided by the authors and unedited

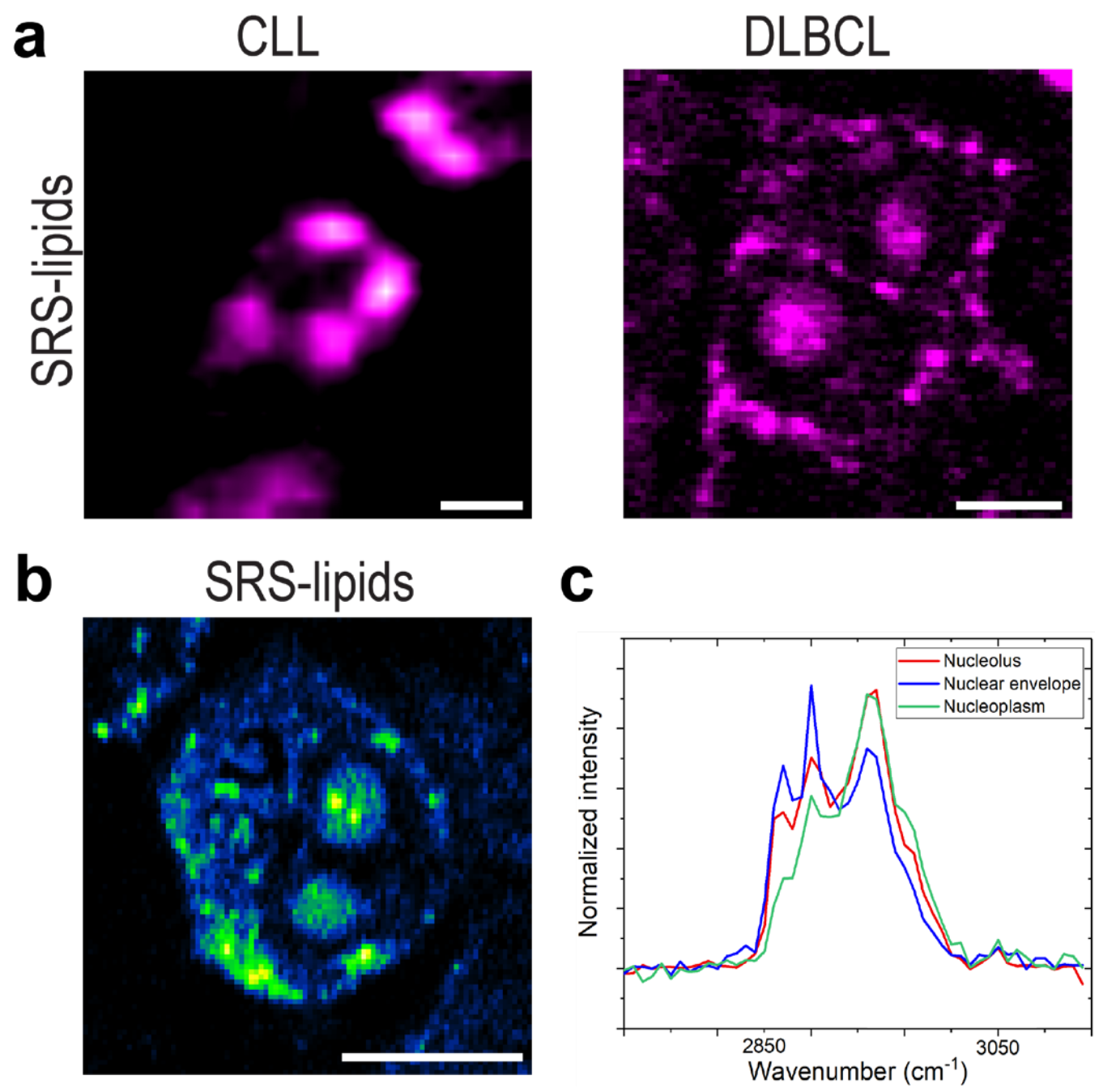

Supplementary Figure 1. REDCAT reveals lipid metabolic reprogramming in tumor cells.

(a) High-magnification and super-resolution SRS images highlighting distinct LD morphology in CLL and DLBCL cells. Scale bar, 5 µm. **(b)** High-magnification and low-exposition SRS image highlighting LDs in nucleolus and perinuclear location of DLBCL cells. Scale bar, 10 µm. **(c)** SRS spectra from different compartments of the nuclei from DLBCL cell.
