# Supplementary material for: All-optical multimodal mapping of single-cell-type-specific metabolic activities via REDCAT
Source: Nat Methods. 2026 Aug 27;23(9):1840–53. doi: 10.1038/s41592-026-03180-0 (PMC13541623; doi:10.1038/s41592-026-03180-0)
Supplement: Supplementary file 2 [file 41592_2026_3180_MOESM2_ESM.pdf]

nature portfolio

Corresponding author(s): Lingyan Shi

Last updated by author(s): Sep 25, 2025

Reporting Summary

Nature Portfolio wishes to improve the reproducibility of the work that we publish. This form provides structure for consistency and transparency in reporting. For further information on Nature Portfolio policies, see our Editorial Policies and the Editorial Policy Checklist.

Statistics

For all statistical analyses, confirm that the following items are present in the figure legend, table legend, main text, or Methods section.

n/a	Confirmed	
☐	☒	The exact sample size (*n*) for each experimental group/condition, given as a discrete number and unit of measurement
☐	☒	A statement on whether measurements were taken from distinct samples or whether the same sample was measured repeatedly
☐	☒	The statistical test(s) used AND whether they are one- or two-sided<br>*Only common tests should be described solely by name; describe more complex techniques in the Methods section.*
☒	☐	A description of all covariates tested
☒	☐	A description of any assumptions or corrections, such as tests of normality and adjustment for multiple comparisons
☐	☒	A full description of the statistical parameters including central tendency (e.g. means) or other basic estimates (e.g. regression coefficient) AND variation (e.g. standard deviation) or associated estimates of uncertainty (e.g. confidence intervals)
☐	☒	For null hypothesis testing, the test statistic (e.g. *F*, *t*, *r*) with confidence intervals, effect sizes, degrees of freedom and *P* value noted<br>*Give P values as exact values whenever suitable.*
☒	☐	For Bayesian analysis, information on the choice of priors and Markov chain Monte Carlo settings
☐	☒	For hierarchical and complex designs, identification of the appropriate level for tests and full reporting of outcomes
☒	☐	Estimates of effect sizes (e.g. Cohen's *d*, Pearson's *r*), indicating how they were calculated

Our web collection on statistics for biologists contains articles on many of the points above.

Software and code

Policy information about availability of computer code

Data collection	N/A
Data analysis	To co-register the modalities, we manually selected a set of visually reliable control points in the SRS and CODEX images. Given the two coordinate arrays, we estimated an optimal affine transformation by least squares using skimage.transform.estimate_transform(), which minimizes the sum of squared distances between matched control points. The SRS image was mapped into the CODEX coordinate system by applying the inverse transform with skimage.transform.warp(), yielding pixel-level alignment suitable for downstream cell-based analysis. Mapped cell centroids were then generated accordingly so that per-cell measurements could be compared one-to-one across SRS and CODEX. At the single-cell level, we computed ratio metrics reflecting metabolic and biochemical state, including NADH/FAD (redox balance), Lipid/Protein (overall lipid enrichment), and Unsaturated-to-Saturated lipid (degree of lipid unsaturation). For each metric, we summarized the upper tail by the 0.8 quantile to capture high-value distributions while remaining robust to occasional extremes. Scatter plots were generated to visualize the spatial distribution of the computed ratios across the tissue. In practice, affine alignment is generally sufficient for fixed sections acquired in close succession, providing a transparent, low-variance correction. Subtle local warping at tissue edges or folds may benefit from higher-order models, which could be worth exploring in cases with pronounced distortion.

For manuscripts utilizing custom algorithms or software that are central to the research but not yet described in published literature, software must be made available to editors and reviewers. We strongly encourage code deposition in a community repository (e.g. GitHub). See the Nature Portfolio guidelines for submitting code & software for further information.

Data

Policy information about availability of data

All manuscripts must include a data availability statement. This statement should provide the following information, where applicable:

- Accession codes, unique identifiers, or web links for publicly available datasets
- A description of any restrictions on data availability
- For clinical datasets or third party data, please ensure that the statement adheres to our policy

Provide your data availability statement here.

Research involving human participants, their data, or biological material

Policy information about studies with human participants or human data. See also policy information about sex, gender (identity/presentation), and sexual orientation and race, ethnicity and racism.

Reporting on sex and gender	N/A
Reporting on race, ethnicity, or other socially relevant groupings	N/A
Population characteristics	N/A
Recruitment	N/A
Ethics oversight	N/A

Note that full information on the approval of the study protocol must also be provided in the manuscript.

Field-specific reporting

Please select the one below that is the best fit for your research. If you are not sure, read the appropriate sections before making your selection.

☒ Life sciences ☐ Behavioural & social sciences ☐ Ecological, evolutionary & environmental sciences

For a reference copy of the document with all sections, see nature.com/documents/nr-reporting-summary-flat.pdf

Life sciences study design

All studies must disclose on these points even when the disclosure is negative.

Sample size	2 sections for healthy lymph node tissue, 1 section for lymphoma tissue, 2 sections for liver tissue
Data exclusions	No data was excluded
Replication	To make sure the lipid phenotype we observed is valid, we used lipid dye and adjunct section to stain the tissue and confirm the lipid accumulation.
Randomization	De-identified archived human lymphoid tissue samples were obtained from Yale Pathology Tissue Services (YPTS). The tissue retrieval and distribution for research were conducted with the approval of the Yale University Institutional Review Board (Approved IRB #1401013259) and oversight by the Tissue Resource Oversight Committee. Written informed consent for participation in any case where identification was collected alongside the specimen, was obtained from patients or their guardians, in accordance with the principles of the Declaration of Helsinki. Each sample was handled in strict compliance with HIPAA regulations, University Research Policies, Pathology Department diagnostic requirements, and Hospital by-laws.
Blinding	Not blinded.

Reporting for specific materials, systems and methods

We require information from authors about some types of materials, experimental systems and methods used in many studies. Here, indicate whether each material, system or method listed is relevant to your study. If you are not sure if a list item applies to your research, read the appropriate section before selecting a response.

Materials & experimental systems

n/a	Involved in the study
☐	☒ Antibodies
☒	☐ Eukaryotic cell lines
☒	☐ Palaeontology and archaeology
☒	☐ Animals and other organisms
☒	☐ Clinical data
☒	☐ Dual use research of concern
☒	☐ Plants

Methods

n/a	Involved in the study
☒	☐ ChIP-seq
☒	☐ Flow cytometry
☒	☐ MRI-based neuroimaging

Antibodies

Antibodies used	Commercial antibodies for lymph node and liver codex experiments were acquired from Akoya
Validation	All antibodies have been validated by Akoya and we did not use any newly synthesized antibody DNA conjugates, and according to NIH criteria, this can be considered as analytically validated. Also, we perform regular quality control tests using a set of standard human tonsil tissue sections prepared and stored in our -80C, and compare IF signal consistency over time and also to Akoya public dataset for Tonsil and literature data for benchmarking.

Plants

Seed stocks	*Report on the source of all seed stocks or other plant material used. If applicable, state the seed stock centre and catalogue number. If plant specimens were collected from the field, describe the collection location, date and sampling procedures.*
Novel plant genotypes	*Describe the methods by which all novel plant genotypes were produced. This includes those generated by transgenic approaches, gene editing, chemical/radiation-based mutagenesis and hybridization. For transgenic lines, describe the transformation method, the number of independent lines analyzed and the generation upon which experiments were performed. For gene-edited lines, describe the editor used, the endogenous sequence targeted for editing, the targeting guide RNA sequence (if applicable) and how the editor was applied.*
Authentication	*Describe any authentication procedures for each seed stock used or novel genotype generated. Describe any experiments used to assess the effect of a mutation and, where applicable, how potential secondary effects (e.g. second site T-DNA insertions, mosiacism, off-target gene editing) were examined.*
